# The X‐ray crystallographic structure of a designed, self‐assembling, and heme‐binding protein

**DOI:** 10.1002/pro.70829

**Published:** 2026-10-27

**Authors:** Iago de Assis Modenez, Véronique Roig‐Zamboni, Philippe Leone, Anabella Ivancich, Vincent L. Pecoraro

**Affiliations:** ^1^ Department of Chemistry University of Michigan Ann Arbor Michigan USA; ^2^ Bioénergétique et Ingénierie des Protéines, Unité Mixte de Recherche UMR 7281, CNRS Aix‐Marseille Université Marseille France; ^3^ Architecture et Fonction des Macromolécules Biologiques, CNRS Aix‐Marseille Université Marseille France; ^4^ Ingénierie des Systèmes Macromoléculaires (UMR 7255), Institut de Microbiologie de la Méditerranée, CNRS Aix‐Marseille Université Marseille France

**Keywords:** alpha‐helical tetramers, coiled coils, cytochrome b, de novo design, heme, metalloprotein

## Abstract

De novo heme‐binding proteins offer powerful minimal scaffolds for elucidating how sequence, oligomerization, and environment rule cofactor coordination, yet high‐resolution structural data for these systems remain scarce. Here, we report the high‐resolution crystal structure of a designed, self‐assembling α‐helical and heme‐binding protein determined at 1.9 Å resolution, that undergoes a pronounced pH‐ and cofactor‐driven reorganization. The structure crystallized in the orthorhombic P2_1_2_1_2_1_ space group (*a* = 44.8 Å, *b* = 46.3 Å, *c* = 101.2 Å), with one tetramer per asymmetric unit and refinement statistics of *R*_fac_/*R*_free_ = 23.5/25.5%. The apo protein folds into a three‐stranded coiled coil, whereas heme binding under strongly basic conditions disrupts register‐specific interactions and drives a major helix shift. This transition yields a four‐stranded coiled coil with an out‐of‐register ABAB arrangement that accommodates two bis‐histidine‐ligated hemes. Cofactors are His‐coordinated via the ε‐nitrogen (Fe‐N_His_ ≈ 2.0 Å), separated by 38 Å (tetrapyrroles edge‐to‐edge) and arranged nearly coplanar with a 67° rotation offset relative to one another. Imidazole dihedral angles (99° and 108°) indicate near‐orthogonal geometry for the His axial ligands to the heme. Crystallization is promoted by a heme‐templated packing in which interactions between macrocycles of neighboring assemblies and several salt‐bridges generate an extended supramolecular lattice. These results demonstrate how heme binding and proton concentration reshape oligomeric topology and promote ordered assembly in minimal protein systems, highlighting the varied structural effects of heme binding and providing principles for stabilizing specific coordination and exploiting cofactor–cofactor interactions to guide high‐order organization in de novo metalloproteins.

## 1 INTRODUCTION

Natural hemoproteins are involved in a remarkable diversity of functions, ranging from electron transport to catalysis and sensing (Poulos, 2014). This diversity is not an inherent property of the heme cofactor alone, but emerges from its integration within a finely tuned protein environment. By controlling redox potential, axial ligation, and substrate accessibility, the protein scaffold enables selective and efficient catalysis. De novo design can provide a minimalistic platform for understanding how sequence, packing, and oligomerization dictate heme binding and stability by reducing the structural complexity inherent to natural proteins (Chalkley et al., 2021; Gibney & Dutton, 1999; Koebke et al., 2022; Modenez et al., 2025; Watkins et al., 2014). In this way, individualized modifications of sequence may be used to examine selectively the impact of charge, hydrogen bonding, hydrophobicity, and so forth. on the properties of the heme. Over the past decades, many designed systems have successfully recapitulated high‐affinity heme binding, yet only a limited number have been structurally resolved at high‐resolution (Ennist et al., 2022; Hou et al., 2025; Hutchins et al., 2023; Kalvet et al., 2023), which is essential to verify design accuracy, heme incorporation, and to guide future rational optimization.

While recent breakthroughs in protein structure prediction, most notably AlphaFold 3 (Abramson et al., 2024), have achieved precision for many canonical peptides, predicted structures can still deviate substantially from crystallographic data, highlighting current limitations of state‐of‐the‐art prediction models. These approaches often struggle to capture the structural plasticity of proteins and peptides, especially when metals, cofactors, or noncanonical coordination environments are involved (Carugo, 2024; Perkins‐Jechow et al., 2025; Terwilliger et al., 2024). This challenge is amplified when cofactors actively drive folding or when peptide structure is coupled to environmental variables such as pH. Consequently, expanding the library of experimentally determined structures is crucial, not only for elucidating cofactor‐driven assembly rules but also for training predictive machine learning algorithms.

There are several known structurally characterized de novo designed heme‐ and porphyrin‐binding proteins. Notable examples are Holo‐MPP1, which stabilizes an abiological Mn‐porphyrin through a penta‐coordinate His ligation (Mann et al., 2021), and HEM_3.C9 that stabilizes a penta‐coordinated heme through Cys ligation with an opened distal pocket (Krishna et al., 2024). Recently, Baker et al (Kalvet et al., 2023). reported a high‐affinity, catalytic heme‐binding enzyme designed with an open coordination site adjacent to a substrate pocket able to perform oxidation and carbene transfer using a His‐pentacoordinated heme. DeGrado and coworkers (Hou et al., 2025) developed a His‐ligated synthetic porphyrin or heme to perform cyclopropanation and Si–H insertion reactions. In both cases, high‐resolution X‐ray crystal structures reveal excellent agreement to the designed model. Other structural contributions are the de novo diheme maquette 4D2 reported by Anderson et al (Hutchins et al., 2023). which has been used to probe redox tuning and modular assembly in *α*‐helical bundles enabling bis‐His coordination, and the miniaturized mimochrome IV bearing a Co(III)‐porphyrin with a bis‐His axial coordination reported by Lombardi and coworkers (di Costanzo et al., 2004). X‐ray crystal structures of more complex multi‐cofactor designed proteins incorporating bis‐His‐ligated heme are known (Ennist et al., 2022). Also, some structures were characterized using solution NMR (Lombardi et al., 2003; Polizzi et al., 2017; Rosenblatt et al., 2003).

De novo designed proteins are typically helical bundles (or more complex loop‐containing architectures) generally exhibiting folds that are largely independent of environmental conditions such as pH. There is no reported crystal structure of a purely self‐assembling and heme‐binding coiled coil. This limitation may arise from the challenges associated with crystallizing self‐associating synthetic protein scaffolds. Therefore, herein we report the crystal structure of a de novo designed and heme‐binding coiled coil from the GRAND (Zastrow & Pecoraro, 2013) family that exhibits an environment‐sensitive conformational switch in aggregation state, heme coordinating residues, and overall assembly under the crystallization conditions. The family of TRI, GRAND and CoilSer peptides adopt two‐stranded coiled coils (2SCC) at pH< 5 and 3SCCs in solution at neutral or basic conditions (Zastrow & Pecoraro, 2013). While CoilSer crystallizes as an antiparallel 3SCC (Lovejoy et al., 1993), it exists in solution as a parallel aggregate. Substitutions of hydrophobic residues at *a* or *d* positions (e.g., coil‐V_*a*_L_*d*_ (Ogihara et al., 1997) or TRIL23H (Tegoni et al., 2012)) yield exclusively parallel apo or mononuclear metal‐bound assemblies. Antiparallel 3‐helical structures with metal‐binding residues have only been achieved in single‐chain polypeptides, such as α_3_DIV (Chakraborty et al., 2011). Upon complexation with heme, a reorganization into a 4SCC (ABAB), composed of two antiparallel dimers, is observed, resulting in a tetrameric assembly containing two hemes (Koebke et al., 2021). If the GRAND peptide sequence is modified to contain both His and Cys putative ligands, the 4SCC bears two penta‐coordinated hemes binding to the His nitrogens at neutral pH, or to the Cys sulfurs at higher pHs, specifically penta‐coordinated thiolate heme at mildly basic conditions (pH∼9.5) and hexa‐coordinated at pH ≥10.5 (Koebke et al., 2021).

Such a pH‐induced switch for a de novo coiled coil system had not been previously reported and demonstrates how subtle modulation of environmental conditions can redirect heme coordination chemistry and global fold of a minimal helical scaffold. Although assemblies corresponding to the heme coordination modes observed at neutral to mildly basic pH (7–10) have not been crystallized, strongly basic conditions (pH >11) favor the formation of a highly stable scaffold in complex with heme. The holo protein prepared under these strongly basic conditions was subsequently subjected to crystallization, yielding a structure that displays a peptide‐heme arrangement arising from an alternative aggregation state accessible only under specific ligation and pH conditions. The resulting high‐resolution X‐ray crystal structure revises the originally proposed antiparallel 4SCC association, revealing an antiparallel 4SCC with an out‐of‐register ABAB arrangement resulting from disruption of register‐specific contacts and a major helix shift of two monomeric helices. This shift is not simply a packing rearrangement but a structural constraint for accommodating two bis‐His heme ligation motifs, each heme being coordinated by His residues from the resulting parallel helices. The structure recapitulates the heme‐binding site found in natural electron transfer proteins and may assist in evaluating the ability of computational methods to predict alternative conformations and multistate protein landscapes. These findings suggest that heme may act as a self‐organizing crystallographic template, with potential implications for de novo design by helping stabilize specific coordination geometries and facilitating crystallization and higher‐order assembly in minimal peptide systems.

## 2 MATERIALS AND METHODS

### 2.1 Peptide synthesis, purification, and heme complexation

Conducted according to our previously reported procedure (Koebke et al., 2021).

### 2.2 Crystallization, data collection, and processing

The holo protein, typically prepared for the 9‐GHz EPR experiments, that is, 1.5 mM monomeric peptide (10 mg.mL^−1^) in complex with 0.5 mM heme (Koebke et al., 2021), final pH 11.4, was concentrated with AMICON Ultra (3 kDa) microconcentrators to a final monomeric peptide concentration of 50 mg.mL^−1^. Crystallization trials (see Figure S1a) were performed using the sitting‐drop vapor‐diffusion method at 293 K in 96‐well Swissci‐3 plates, with the Nextal AmSO4 screen (Caliber scientific) using Robot Evo150 (Tecan) and Mosquito robots (SPTLabtech) to fill‐in the plates and disperse the drops, respectively. Suitable crystals, that grew using the crystallization solution of 2.0 M NaCl and 2.0 M ammonium sulfate (pH = 5.04), were mounted in cryo‐loops (Hampton CrystalCap Magnetic) and briefly soaked in the crystallization solution and 25% (v/v) glycerol before freezing in LN_2_. When mixing the protein in 0.25 M TRIS maleate buffer pH 11.4 with the crystallization solution pH 5.04 in a 3:1 volume ratio, the resulting pH of the *sample in solution* was 9.0.

Single‐wavelength anomalous dispersion (SAD) data were collected from an analogous heme‐loaded peptide of GRW‐L16CL30H to 2.0 Å resolution, while native diffraction data of holo‐GRW‐L16CL30H were collected to 1.9 Å resolution on the beamline Proxima‐1 at the Soleil synchrotron (Paris, France). The data sets were integrated with XDS (Kabsch, 2010) and scaled with SCALA (Collaborative Computational Project, N. 4, 1994) from the CCP4 suite (Collaborative Computational Project, N. 4, 1994). Heavy atom substructure determination of the analogous heme‐loaded peptide, phase calculations, density modification and automatic model building were performed using HySS (McCoy et al., 2007), Phaser (McCoy et al., 2007), Parrot (Cowtan, 2006), and Buccaneer (Cowtan, 2006), respectively, as implemented in the Phaser SAD pipeline from the CCP4 suite. The partial model obtained was completed manually in COOT (Emsley & Cowtan, 2004), and subsequently used as a model for molecular replacement with Phaser to solve the structure of holo‐GRW‐L16CL30H. Refinement, correction, and validation of the structures were performed with autoBUSTER (Chen et al., 2010), COOT, and Molprobity (Chen et al., 2010), respectively. Data collection and refinement statistics are reported in Table 1, and representative electron‐density maps are displayed in Figures S1b and S8.

**TABLE 1:** Data collection and refinement statistics.

Data collection	
Space group	P2_1_2_1_2_1_
*a*, *b*, *c* (Å)	44.8, 46.3, 101.2
*α*, *β*, *γ* (°)	90, 90, 90
Resolution (Å)^a^	30–1.9 (2.0–1.9)
Unique reflections^a^	17,241 (2461)
Redundancy^a^	13.3 (13.9)
Completeness (%)^a^	99.9 (100.0)
*I*/σ^a^	12.1 (0.9)
*R*_meas_ (%)^a^	8.7 (300.1)
CC1/2	0.999 (0.647)
Mosaicity (°)	0.15

Refinement and model quality	
Resolution (Å)	30.0–1.9
Reflections^a^	17,194
*R*_fac_/*R*_free_ (%)	23.5/25.5
Number of atoms	
Protein/heme/ligand/ion/water	1150/86/6/5/51
B‐factors (Å^2^)	
Protein/heme/ligand/ion/water	70.0/47.0/83.5/71.4
RMSD	
Bond (Å)/angle (°)	0.009/0.99
Ramachandran (%)	
Most favored	97.7
Allowed regions	1.5
Outliers	0.8
PDB accession code	32MQ

^a^ Values in parenthesis are for the highest‐resolution shell.

### 2.3 Structure analysis

Structural analyses were performed using PyMol with custom Python scripts and programs from the CCP4 suite. Structural superpositions and root‐mean‐square deviation (RMSD) calculations were carried out using least‐squares fitting of Cα atoms unless otherwise stated. Interatomic distances, geometric parameters, and side‐chain dihedral angles were measured in PyMol. Buried surface areas were calculated from the difference between the accessible side chain surface areas of the tetramer and of the individual monomers using ArealMol of the CCP4 suite (Winn et al., 2011). Electrostatic mapping was calculated using Adaptive Poisson–Boltzmann Solver (APBS) PyMol built‐in function. Molecular graphics and structural figures were generated either using PyMol or ChimeraX.

## 3 RESULTS AND DISCUSSION

### 3.1 Crystal structure and packing

Vapor‐diffusion crystallization of GRW‐L16CL30H in complex with heme resulted in elongated, dark red crystals (Figure S1b) belonging to an orthorhombic crystal form (P2_1_2_1_2_1_ space group) that diffracted synchrotron X‐rays to 1.9 Å resolution (Table 1). The resulting electron‐density map is well defined and supports confident model interpretation (Figures S1b and S8).

The peptide sequence is Ac‐GWKALEEKLKALEEKCKALEEKLKALEEK**H**KALEEKG‐NH_2_. For three of the four polypeptide chains, 35 residues were modeled, while the fourth chain included 33 residues, covering five heptads in length. The final model includes a single tetramer, 51 water molecules, 5 chloride ions, and 2 hemes in the asymmetric unit, and was refined to satisfactory agreement values (*R*_fac_/*R*_free_ = 23.5/25.5%). It adopts a left‐handed superhelix consisting of four α‐helical monomers, that cross each other at a crossing angle of approximately 20.2° to form a tetramer, with an out‐of‐register ABAB arrangement that accommodates two bis‐histidine ligated hemes (Figure 1).

**FIGURE 1:**
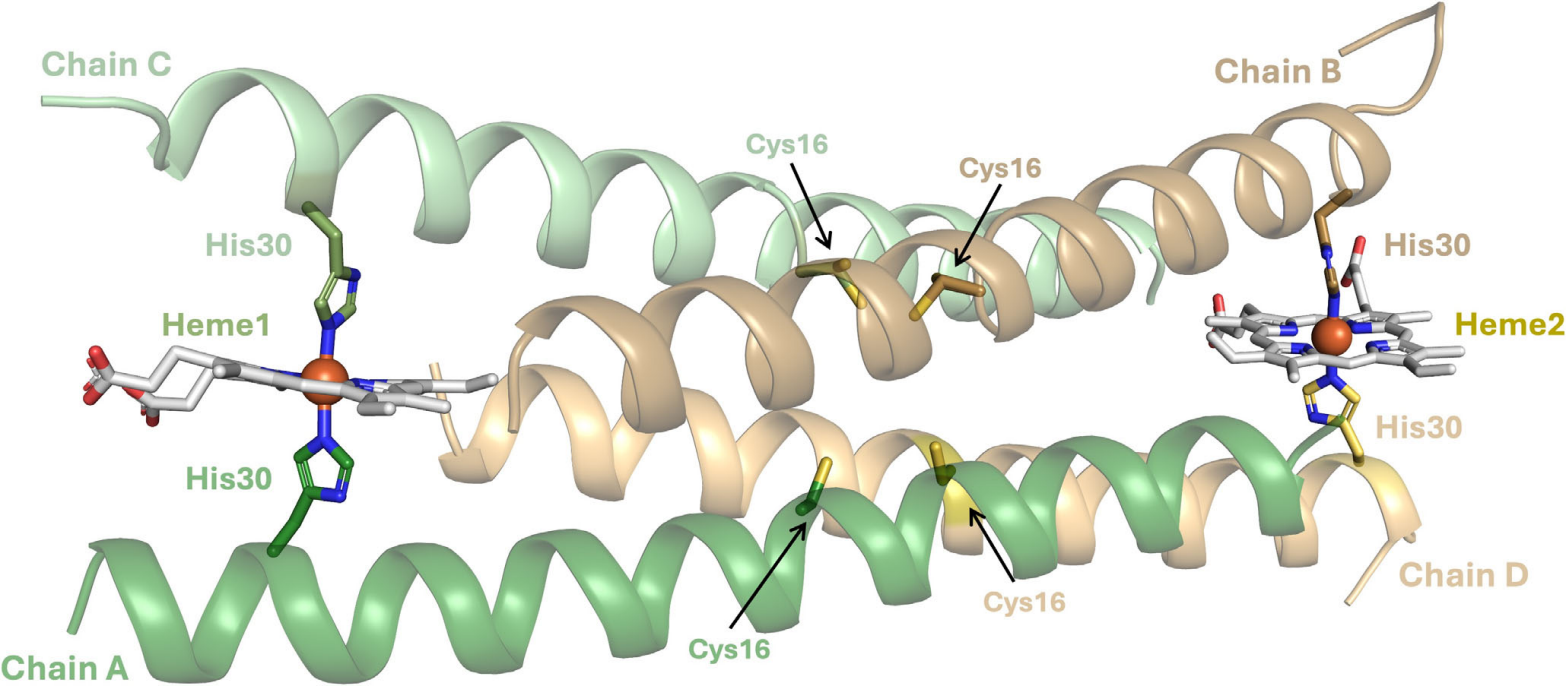
Crystallographic structure of GRW‐L16CL30H protein in complex with hemes (PDB code 32MQ). Ribbon representation of the coiled coil backbone. Parallel chains A and C are shown in dark and light green, respectively, while parallel chains B and D are show in dark and light brown, respectively. Chains A and B are closer to the viewer. The heme axial ligands (His30) are depicted as sticks and colored according to their respective chains (green for A and C; and brown for B and D). The heme cofactors are shown as sticks and the iron ion as a sphere. The four Cys16 residues are also depicted as sticks.

The superhelix forms a cylinder with an overall diameter of ∼16 Å and length of 65 Å (Figure 2a). The average interhelical distances between the axes of the diagonal (parallel) helices are 14.1 and 15.6 Å, whereas those between adjacent (antiparallel) helices are 6.0 and 13.6 Å on one side and 9.0 and 12.7 Å on the other (Figure 2b). Backbone superposition of the four helices reveals a high degree of structural similarity across the assembly, with the largest deviations occurring at their ends. Pairwise C_α_ RMSD values range from 0.09 to 0.99 Å, with an average of 0.69 Å. Chains A and D exhibit near‐identical conformations (RMSD = 0.09 Å), while chains B and C also closely match (RMSD = 0.42 Å), consistent with an approximate C_2_ symmetry relating helix pairs. Superpositions between adjacent and opposing helices yield slightly higher, but still low RMSD values (0.83–0.99 Å).

**FIGURE 2:**
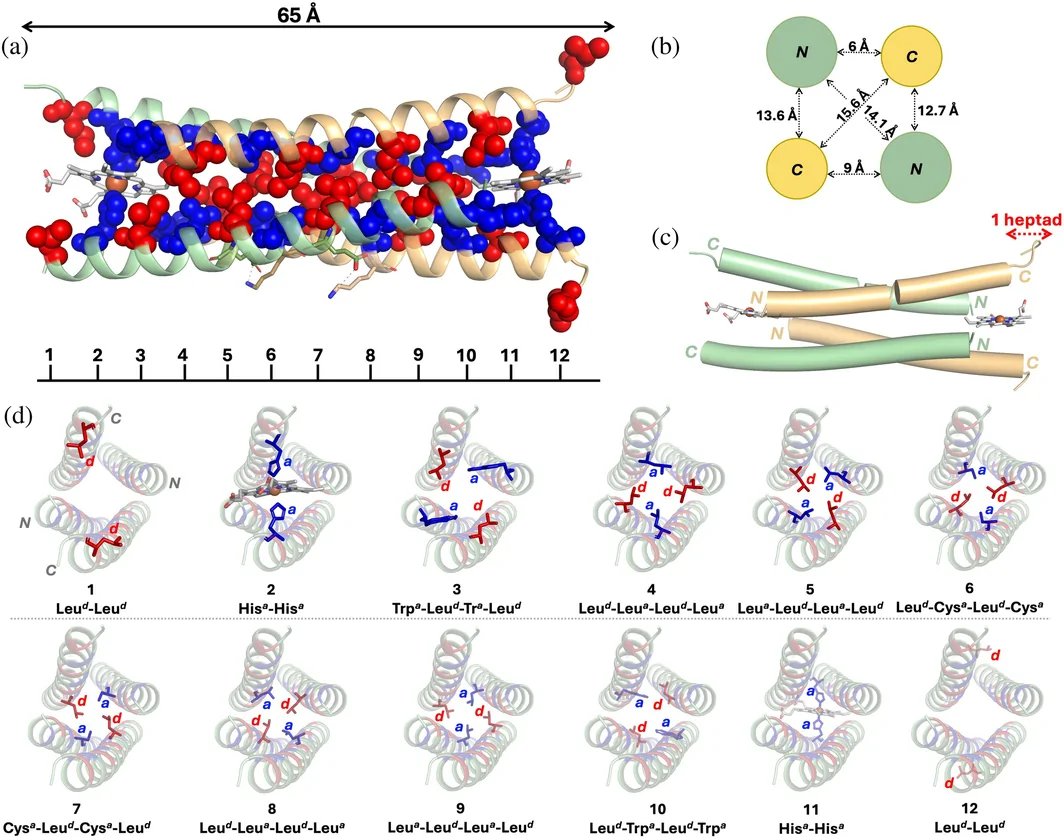
Core packing of holo‐GRW‐L16CL30H. (a) Coiled coil hydrophobic layers. Spheres of the side chains of *a* (blue) and *d* (red) layers on a ribbon backbone are shown. Layer number, counted from the N‐ to C‐terminus, are indicated. (b) Schematic axial view of the tetramer highlighting the distances between adjacent and diagonal chains. (c) Cylinder representation of the four‐chain backbone illustrating the heptad register shift. The N‐ and C‐termini are indicated, and the two heme cofactors are shown as sticks. (d) Geometry of individual hydrophobic layers. Stick representations of each core layer are shown. The *a* and *d* positions are colored as in panel (a). Layer numbers and corresponding residues are also indicated.

Heme‐heme contacts between symmetry‐related tetramers contribute to the crystal packing, generating an extended supramolecular lattice (Figure S2). In addition, several salt bridges (Glu➔Lys) and hydrogen‐bonding interactions contribute to the packing (see Figure S3 and Table S1).

The bis‐His coordination observed in the crystal was not detected in our previous spectroscopic studies in solution, in which hemes showed His30 as axial ligand for pH< 9 and Cys16 for higher pHs (Koebke et al., 2021). Mixing the holo protein at pH 11.4 with the acidic crystallization solution may have decreased the pH, yet the actual pH of the crystallized protein is unknown. The crystallization may have stabilized alternative coordination or conformational states that, due to their low proportion, were not detected in the EPR spectrum in solution; hence, the structure cannot be directly assigned to the equilibrium state for an estimated pH 9.0 in solution. The bis‐His state may reflect sample history, kinetic trapping, and/or crystallization‐induced stabilization, but the present data do not distinguish between these possibilities.

### 3.2 Hydrophobic core packing

Despite the high structural similarity between the chains, the helical register is not fully conserved: chains B and D are slip‐stacked relative to chains A and C by approximately one full heptad repeat (Figure 2c). In this arrangement, chains A and C are parallel and opposed to each other, as are chains B and D. This heptad offset disrupts interchain interactions within the tetramer, leading to local unwinding and adoption of a more extended conformation. This out‐of‐register organization positions the two hemes for bis‐His coordination and significantly influences the core packing and interhelical interactions within the tetramer (Figure S4). The tetramer adopts an organization in which the *a* and *d* positions form stacked layers along the superhelical axis (Figure 2a). Eight layers are symmetric with respect to residue identity (two Leu‐Leu layers, two His‐His layers, and four Leu‐Leu‐Leu‐Leu layers), while the remaining four are asymmetric (two Trp‐Leu‐Trp‐Leu layers and two Leu‐Cys‐Leu‐Cys layers). However, the identities of residues forming individual hydrophobic layers differ from those predicted for a canonical antiparallel tetramer, which would result in two layers formed by residues Leu9, Leu33, Leu12, and His30. In the structure reported here, the same two layers are instead formed by Leu9, Leu19, Leu12, and Cys16 (compare Figure S4 with S14).

The slip‐stacking of two of the chains introduces substantial differences in packing between the central layers to those near the strand termini (Figure 2d). In the middle region (layers 4–9), each layer consists of two diagonally opposed *a‐a′* residues and two *d‐d′* residues, generating a four‐residue hydrophobic layer that interacts in a manner analogous to the classical knobs‐into‐holes packing within the quaternary core. Consistent with this arrangement, most residues occupying the *a* and *d* positions in these layers adopt canonical Leu rotamers (𝜒_1_ ≈ −60° to −80° and 𝜒_2_ ≈ 170° to 180°), corresponding to the gauche^−^/trans (g^−^/t) conformation that directs the side chains toward the interior of the coiled coil and facilitates hydrophobic packing. Some residues, however, exhibit deviations from this geometry, most notably Leu9 in chains B and C, Leu12 across multiple chains, and Leu19 in all four helices, which adopt alternative trans/gauche^+^ (t/g^+^) rotamers. Additional variability is observed for Leu33 in chains C and D (Table S2).

Near the terminal regions (layers 1–2 and 11–12), each layer contains only two diagonally opposed *a‐a′* or *d‐d′* residues, disrupting the original knobs‐into‐holes packing geometry. The core geometry in layers 3 and 10 deviates from the classical arrangement: the *a* and *d* residues are oriented outward, away from the core, rather than pointing inward as in the central layers. The relative arrangement of the individual chains also differs from that in the central region of the coiled coil. Instead of the symmetrical square‐like arrangement of chains A, B, C, and D observed in the central layers, the terminal portions of the helices adopt a more linear arrangement in which chains A and C, and chains B and D, form opposing pairs (Figure S5).

Analysis of buried surface area by heptad position reveals that the middle region forms a canonical coiled coil core, with the *a* and *d* positions exhibiting 93% and 83% mean burial, respectively (Tables 2, S3. and Figure S6). On the other hand, the terminal region displays a pronounced reduction in hydrophobic core packing, with burial at the *a* and *d* positions decreasing to 48% and 21%, respectively. Notably, His30, located at an *a* position within the C‐terminal, exhibits substantially reduced burial (34%) relative to canonical core *a* residues, suggesting localized opening of the superhelical core that facilitates solvent accessibility to the coordinated heme (Figure S7). Residues at the *e* and *g* positions also exhibit moderate burial (57% and 54%, respectively), while *b* and *f* positions along the exterior surface remain highly solvent exposed. Approximately 35% of the total isolated monomer solvent‐accessible surface area (3833 ± 131 Å^2^, on average) becomes buried upon tetramer formation.

**TABLE 2:** Coiled coils parameters.

Average buried surface area by heptad position (layers 4–9) (%)	
*a*	93
*b*	3
*c*	26
*d*	83
*e*	57
*f*	0
*g*	54

*α*‐helix parameter	
*α*‐helix radius, *r*_1_	2.34 ± 0.02 Å
Angular frequency, *ω*_1_	97.3 ± 2.1°/residue
Rise per residue, *h*	1.49 ± 0.03 Å
Residues per *α*‐helical turn, *n*	3.7 ± 0.08 residues/turn

Superhelix parameter	
Supercoil radius, *r*_0_	7.0 Å
Tilt angle relative to superhelical axis, *α*	13.3°
Supercoil pitch, *P*	186 Å
Translation of the supercoiled helix along the superhelical axis, *Z*_trans_	1.44 ± 0.03 Å/residue
Residues per superhelix turn, *n*	127 ± 2 residues/turn

*Note*: Values for the individual chains (A, B, C, and D) are in Table S4.

The formation of two bis‐His‐coordinated hemes also induces a complete rearrangement of the Cys16 residues, generating a sulfur‐rich tetracysteinyl pocket reminiscent of rubredoxin‐like coordination environments (Tebo et al., 2018). In chains A and D, Cys16 is fully ordered and modeled in a single conformation with unit occupancy (Figure S8). In contrast, chains B and C exhibit alternate conformations extending from the C_α_ position through the cysteine side chain, with occupancies of 0.63/0.37 and 0.54/0.46, respectively. Because the disorder originates at the C_α_ position and propagates though the side chain, the observed heterogeneity reflects a localized conformational rearrangement rather than a simple terminal side chain rotameric fluctuation. These differences among subunits likely arise from subtle asymmetries in crystal packing or local intermolecular interactions. The average S–S distance between those of diagonal helices is ∼4.6 Å, whereas that between those of adjacent helices is ∼7.1 and ∼8.2 Å.

### 3.3 Electrostatic interactions and H‐bonding

The holo‐GRW‐L16CL30H crystal structure is stabilized by an intermolecular salt bridge between the side‐chain carboxylate of Glu20 (*e*) in chain A and the side‐chain ammonium group of Lys10 (*b*) in chain D. Glu20 adopts two conformations, each with an occupancy of 0.5, and both conformers maintain the interaction with Lys10. In addition, a water‐mediated hydrogen bond is observed between Lys3 (*b*) of chain A and Glu27 (*e*) of chain B (Figures 3a and S4).

**FIGURE 3:**
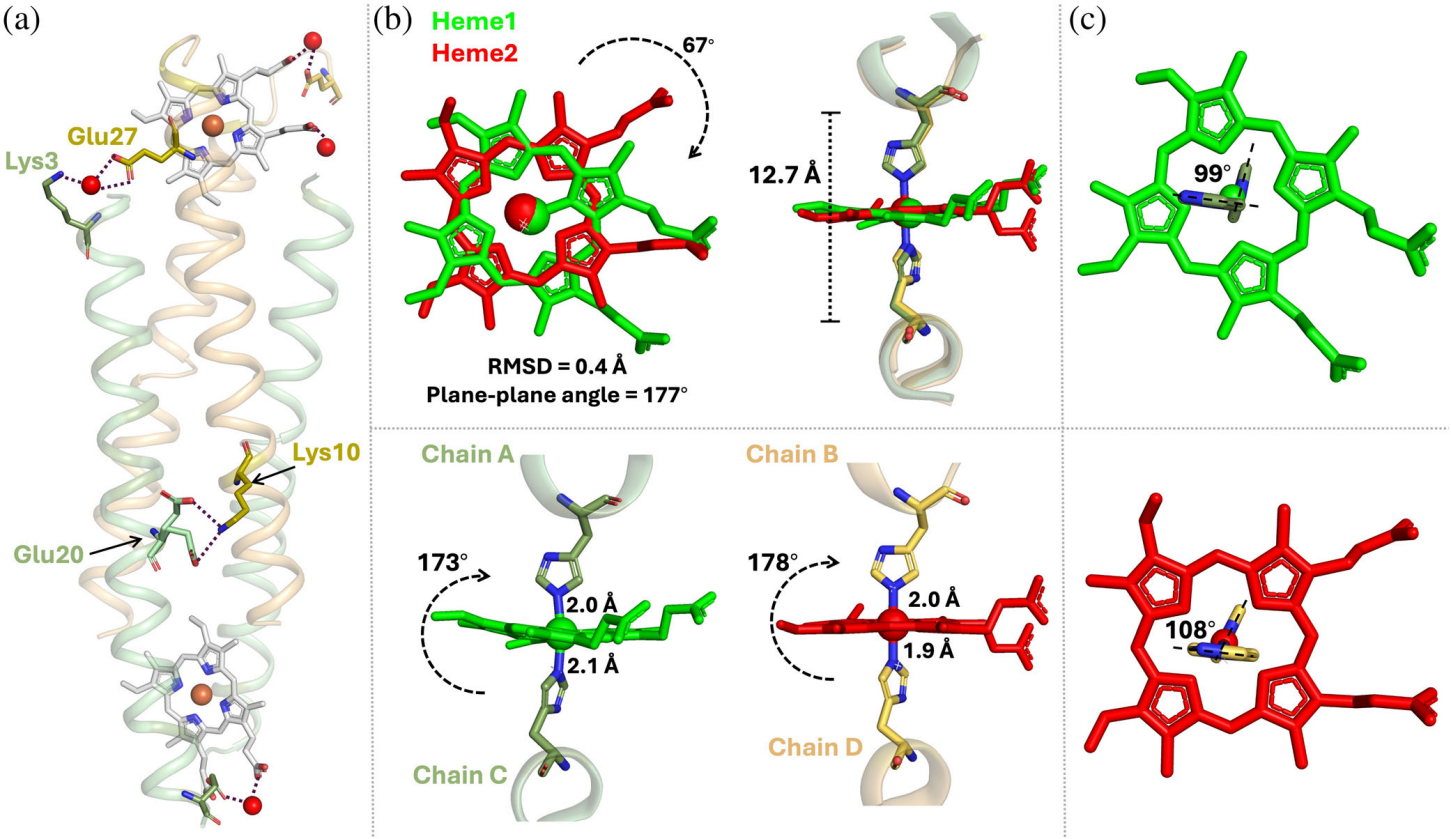
Interfacial interactions and heme cofactors parameters. (a) Salt bridges and water‐mediated hydrogen bonds formed between charged residues across adjacent helices contribute to stabilization of the tetramer interface. Protein backbones are shown as ribbons, interacting as sticks, and water molecules are red spheres. Dashed lines indicate hydrogen‐bonding and electrostatic interactions at the interface of neighboring chains. (b) Superposition of heme1 (green‐colored sticks) and heme2 (red‐colored sticks) cofactors illustrating their high structural similarity (RMSD = 0.4 Å). The porphyrin planes are nearly parallel while exhibiting a rotational offset around the Fe–Fe axis. Coordination environment of the hemes iron showing axial ligation by two His residues is also shown. Fe‐N_ε_(His) bond distances are ∼2.0 Å, and the ligands approach the porphyrin plane from opposite sides with near‐linear geometry. The coordinating imidazole rings are arranged in a near‐orthogonal orientation. (c) Ball and stick representation of heme1 (green), heme2 (red), and their respective His30 imidazole ligands, viewed along the axial bond direction. The imidazole ring planes dihedral angle for both hemes is shown.

A regular pattern of backbone intrachain hydrogen bonds (CO_*i*_ ← HN_*i*+4_) is observed in all four chains, with an average H‐bond distance of 3.02 ± 0.03 Å for chain A (first carbonyl group involved is that of Gly1 and the last NH is that of Glu35), 2.92 ± 0.03 Å for chain B (Gly1‐Lys31), 3.02 ± 0.02 Å for chain C (Gly1‐Glu34), and 2.90 ± 0.03 Å for chain D (Gly1‐Leu33).

Electrostatic surface potentials calculated using APBS reveal a pronounced segregation of positive and negative regions across the tetramer (Figure S9). A continuous positively charged patch is observed along the *g/g′* interfaces formed predominantly by Lys residues between antiparallel chains A‐B and C‐D, whereas negatively charged patches corresponding primarily to Glu residues at the *e/e′* positions are located at the A‐D and B‐C interfaces. In addition, although mostly solvent‐exposed, the heme cofactor resides within a mixed electrostatic environment where positively charged residues flank the propionate groups, potentially contributing to cofactor accommodation within the tetrameric bundle. Further analysis of the inter‐tetramer contacts in the crystal lattice reveals the formation of channels approximately 30 Å in diameter (Figure S10). These channels are lined by alternating positively and negatively charged surface patches, generating a mixed electrostatic environment along their length. They likely act in concert with the heme–heme contacts between neighboring tetramers, collectively promoting formation and stabilization of the extended lattice.

### 3.4 Hemes

The two heme cofactors are each positioned between a pair of parallel helices: heme1 between chains A and C and heme2 between chains B and D. Both hemes are coordinated by the ε‐nitrogens of the imidazole ring of the His30 residues from each pair of helices. (Figure 3b), which can be viewed as a structural mimic of naturally occurring hemoproteins involved in electron transfer, such as the cytochrome *b* subunits of mitochondrial complexes (Berry & Walker, 2008). The Fe‐N_His_ bond lengths are approximately 2.0 Å on average for all four coordinating His, and the edge‐to‐edge distance between the conjugated tetrapyrrole rings for the two hemes is 38 Å. Superposition of the coordinated hemes reveals a high structural similarity, with an RMSD of 0.4 Å. The coordinating His residues adopt a well‐defined 𝜒_1_/𝜒_2_ = g^−^/g^+^ rotameric state (𝜒_1_ ≈ −80° and 𝜒_2_ ≈ +70°). They adopt nearly ideal linear axial coordination, with the His(N_ε_)‐Fe‐His(N_ε_) angles measuring 173° for heme1 and 178° for heme2, indicating one ligand approaches from above the porphyrin plane and the other from below. The dihedral angle between the imidazole ring planes of the axial His ligands are 99° and 108° for heme1 and heme2, respectively, indicating a near‐orthogonal arrangement (Figure 3c, Table 3). The heme planes themselves are closely coplanar, with a plane‐plane angle of 177°, while the two hemes exhibit a relative rotational offset of 67°. The C_α_–C_α_ distances between the axial His residues are approximately 12.7 Å for the two heme sites. The porphyrin macrocycle of heme1 displays a modest, ruffled distortion, while heme2 adopts an essentially planar conformation. Only minor differences are observed, reflecting subtle local packing asymmetry without substantial electronic perturbation. Such cofactor geometry is consistent with constrained bis‐His coordination generally found in natural cytochromes and likely minimizes steric repulsion between axial ligands and the porphyrin plane while maintaining stable Fe–N bonding (Walker, 2004).

**TABLE 3:** Comparison of the heme coordination parameters for selected de novo designed antiparallel tetramers and natural bis‐His hemoproteins.

De novo proteins	Heme	Fe‐N_His_ length	His‐His 𝜙	References
This work	2 hemes b	2.0 Å	Heme1 99° Heme2 108°	‐
PDB code 5VJT	Heme b	1.9 Å	145°	(Ennist et al., 2022)
PDB code 7AH0	2 hemes b	2.1 Å	Heme1 112° Heme2 115°	(Hutchins et al., 2023)

Natural proteins				
PDB code 1Q1F	Heme b	2.0 Å	119°	(Vallone et al., 2004)
PDB code 1URV	Heme b	2.2 Å	112°	(de Sanctis et al., 2004)
PDB code 1AQA	Heme b	2.0 Å	68°	(Baker et al., 1999)
PDB code 1QHU	Heme b	2.2 Å	172°	(Baker et al., 1999)

The heme pockets are partially hydrated (Figure S7), with three water molecules mediating hydrogen bond interactions between the propionate groups and nearby residues (Figure 3a), which likely contributes to local stabilization and proper cofactor orientation. Specifically, one water bridges the propionate of heme1 to Glu34 (chain A), while two waters perform analogous bridging between heme2 and Glu35 (chain B) and the backbone NH of Trp2 residue (chain C), respectively. A direct interaction is observed between one of the propionate groups and Lys29 (chain D). These interactions, at distances of 3.4–3.6 Å, provide partial anchoring of the hemes within the tetrameric assembly. In addition, the indole ring of the Trp2 residues adopts a slipped parallel orientation relative to the porphyrin macrocycle, with an interplanar separation of approximately 3.5–3.7 Å, consistent with favorable 𝜋‐stacking interactions (Figure S11).

The change in oligomerization state and the one‐heptad registry shift (see Figure 2c) likely arise from the combined geometric and energetic constraints imposed by bis‐His heme coordination and interhelical electrostatic interactions, enabling reorganization of the coiled coil. Formation of the bis‐His antiparallel tetramer involves loss of the preceding ferric heme‐thiolate interactions of the 4SCC (see Figure S14) (+20–40 kcal/mol), offset by stabilization from tetramer assembly and two bis‐His heme ligations (∼ − 45 kcal/mol total). The basic conditions may facilitate access to this alternative assembly pathway by increasing the conformational flexibility of the peptide, thereby enabling partial helix unwinding; similar unfolding has been previously reported (Hou et al., 2025), and lowering the energetic barriers between distinct coiled coil registries. In this context, the heme cofactors may act as structural organizing elements that guide the peptide into a specific oligomeric state through Fe‐N(His) coordination.

### 3.5 Comparison with de novo antiparallel tetramers and natural hemoproteins

Comparison with selected reported de novo coiled coil and helical bundle designs shows that our structure exhibits larger average interhelical separation (15 Å diagonally and 7.5–13 Å adjacently), exceeding those typically observed in bundle architectures (∼12 Å diagonally and ∼6.7–9.6 Å adjacently) (Table 4). Consistent with this expanded geometry, the per‐residue average accessible surface area (ASA, 70 Å^2^) is higher than that of most apo and heme‐bound tetramers (36–65 Å^2^), indicating a more solvent‐exposed and less compact structure resulted from the helix shift to accommodate the bis‐His‐ligated hemes. The number of interhelical salt bridges is lower than in related systems, suggesting that these interactions do not significantly contribute to the stability of the present coiled‐coil assembly. In accordance, the remarked influence of heme is evident when comparing the present structure with the apo 3SCC coiled coil (PDB code 7N2Y). All structural parameters change upon heme binding. Heme alters peptide assembly in two ways: first, by converting a parallel 3SCC into an antiparallel 4SCC across all pH ranges, and second, by inducing slip stacking at basic pH. These observations support a model in which bis‐His heme coordination plays a dominant structure‐directing role, compensating for suboptimal packing and enabling formation of a well‐defined tetramer.

**TABLE 4:** Comparison of structural parameters with selected de novo structures.

PDB	Oligomer/fold	Cofactor/type of ligation	Average interhelical separation	Salt bridges	Per‐residue average ASA	References
PDB code 7N2Y	Trimer/coiled coil	Apo	Diagonal 12 Å Adjacent 7.5 Å	12	65 Å^2^	(Pinter et al., 2021)
This work	Tetramer/coiled coil	Heme b/bis‐His	Diagonal 15 Å Adjacent 7.5 Å and 13 Å	1	70 Å^2^	‐
PDB code 5VJT	Tetramer/bundle	Heme b/bis‐His	Diagonal 12.1 Å Adjacent 7.0 and 11 Å	3	57 Å^2^	(Ennist et al., 2022)
PDB code 7AH0	Tetramer/bundle	Heme b/bis‐His	Diagonal 16.9 Å Adjacent 5.8 and 13.2 Å	0	59 Å^2^	(Hutchins et al., 2023)
9CTE	Tetramer/bundle	Synthetic Fe‐porphyrin/His‐H_2_O	Diagonal 12.6 Å Adjacent 7.5 and 9.1 Å	3	65 Å^2^	(Hou et al., 2025)
PDB code 7JRQ	Tetramer/bundle	Synthetic Mn‐porphyrin/His‐H_2_O	Diagonal 14 Å Adjacent 6.5 Å and 8.2 Å	3	63 Å^2^	(Mann et al., 2021)
PDB code 8A3J	Tetramer/coiled coil	Apo	Diagonal 10 Å Adjacent 8.0 and 6.1 Å	3	51 Å^2^	(Naudin et al., 2022)
PDB code 1W5H	Tetramer/bundle	Apo	Diagonal 12.6 Å Adjacent 7.8 and 9.5 Å	4	36 Å^2^	(Yadav et al., 2006)
PDB code 2B1F	Tetramer/coiled coil	Apo	Diagonal 10.7 Å Adjacent 5.5 and 10.8 Å	4	59 Å^2^	(Deng et al., 2006)

Our design binds two heme b molecules with Fe‐N(His) distances comparable to previously reported de novo and natural hemoproteins structures (Table 3). In contrast, the His‐His dihedral angles (99° and 108°) fall within the narrower range typically observed for designed systems (∼100–120°), but are more restricted than those found in natural proteins, which span a broader distribution (∼68–172°), although ligands in bis‐His hemes are found more frequently perpendicular rather than parallel to each other (Fufezan et al., 2008).

## 4 CONCLUSION

Herein, we report the high‐resolution crystal structure of a heme‐binding de novo designed peptide at basic pH, demonstrating how bis‐His coordinated heme cofactors direct the assembly of a coiled coil into a well‐defined antiparallel tetramer. This behavior reveals the structural plasticity of a single designed sequence, which has previously been shown to function as a pH‐dependent structural mimic of peroxidases, chloroperoxidases, and cytochrome P450 monooxygenases within a minimal scaffold. The ability of a single sequence to adopt multiple oligomeric states, altered hydrophobic packing, and helix registries underscores the delicate energetic balance governing coiled coil formation. In this context, the heme cofactors may act as a structural determinant, stabilizing the assembly while dictating oligomeric state, helix registry, and supramolecular organization, and adopting geometries consistent with natural electron transfer proteins, yet under basic conditions in the holo GRW‐L16CL30H scaffold.

These findings pave the way for the rational design of redox‐active artificial metalloproteins while also providing a potential future test case for computational approaches aimed at predicting alternative oligomeric states and structural plasticity in designed proteins, which often struggle to accurately model metal‐binding sites and alternative registry arrangements. The registry shift observed in the present tetramer is unusual among designed heme proteins and exemplifies the type of structural variability that remains difficult to predict from sequence evaluation alone. Future work will explore the use of bis‐His coordinated hemes in these 4SCCs as engineered electron transfer sites.

## AUTHOR CONTRIBUTIONS

**Iago de Assis Modenez:** Investigation; writing – original draft; methodology; writing – review and editing; formal analysis; data curation. **Véronique Roig‐Zamboni:** Investigation; writing – review and editing; methodology. **Philippe Leone:** Investigation; writing – review and editing; methodology; validation; formal analysis; data curation. **Anabella Ivancich:** Conceptualization; investigation; funding acquisition; writing – review and editing; methodology; validation; project administration; data curation; supervision; resources. **Vincent L. Pecoraro:** Conceptualization; funding acquisition; writing – review and editing; writing – original draft; resources; formal analysis; project administration; supervision.

## CONFLICT OF INTEREST STATEMENT

The authors declare no competing financial interest.

## Supporting information


**Table S1.** Interactions between the tetramer and neighboring chains interconnecting the tetramers to generate the three‐dimensional crystal lattice (4 Å‐cutoff ).
**Table S2**. Side chain rotamers (𝜒_1_ and 𝜒_2_) for residues at *a* and *d* position.
**Table S3**. Average buried surface area by heptad position for the core layers (4–9), termini ends (layer 1–2 and 11–12) and the whole tetramer (layers 1–12).
**Table S4**. Coiled coil parameters.
**Table S5**. Sequence of relevant peptides discussed in this study.
**Figure S1a**. Crystals of holo‐GRW‐L16CL30H obtained using different crystallization conditions.
**Figure S1b**. Crystals of holo‐GRW‐L16CL30H diffracted up to 1.9 Å resolution. Optical image of crystals of holo‐GRW‐L16CL30H (Left). Representative electron‐density map (2F0Fc, contoured at 1𝜎) around the heme1 (Middle) and heme2 (Right).
**Figure S2**. Porphyrin–porphyrin contacts between symmetry‐related tetramers in the crystal lattice. (Top) Closest intermolecular contact between neighboring heme cofactors, occurring between a vinyl carbon of one heme and a carbon atom of the imidazole ring from a neighboring tetramer. (Bottom) Top view of two symmetry‐related tetramers highlighting the offset arrangement of the heme cofactors between the tetramers.
**Figure S3**. Heme–heme and salt bridge contacts between symmetry‐related tetramers. (Top) Heme‐heme contacts generate an approximately linear arrangement of tetramers across the crystal lattice. (Bottom) Salt‐bridges between Glu and Lys residues connect Tetramer 1 (light brown) with neighboring tetramers. In the left panel, heme1 is shown, highlighting salt bridges between this tetramer and three neighboring tetramers (blue, light green, and salmon). In the right panel, heme 2 is shown, where several salt bridges connect this tetramer with two neighboring tetramers (light blue and light gray).
**Figure S4**. Helical wheel diagram for antiparallel holo‐GRW‐L16CL30H. Two peptide helices (light green) extend from the N‐terminus (emerging from the page) to the C‐terminus (projecting into the page), while the other two (light yellow) extend from the C‐terminus (emerging from the page) to the N‐terminus (projecting into the page) to form an out‐of‐register ABAB arrangement. Each hydrophobic layer is formed by interactions among four hydrophobic side chains of *a*‐*d*‐*a*′*‐d*′ or *d‐a‐d*′*‐a*′. The core interaction for the second heptad (from the N‐terminal) is exemplified: Leu9‐Leu19‐Leu9‐Leu19 (dotted orange line) and Leu12‐Cys16‐Leu12‐Cys16 (dotted brown line). The interhelical salt bridge between K10 and E20 is shown as a dotted red trace, while the water‐mediated (red sphere) hydrogen bond between K3 and E27 is shown as dotted black traces.
**Figure S5**. Cylinder representation of holo‐GRW‐L16CL30H. Cross‐sections of the helical arrangements are shown for the center and ends of the coils.
**Figure S6**. Solvent exposure mapped onto the tetramer structure. (Top) Average residue solvent exposure calculated using ArealMol from CCP4 suite and mapped onto the structure. The protein is colored according to normalized solvent accessibility, ranging from white (least exposed, *b* = 0) to dark blue (most exposed, *b* = 1). (Bottom) Average buried surface area (%) bar chart per heptad position for tetramer core, termini, and total.
**Figure S7**. Water accessibility to the tetramer. A mesh surface surrounding the tetramer highlights regions of increased hydration near the heme cofactors. Most of the heme macrocycle remains solvent‐exposed. Protein backbones are shown as ribbon, the coordinating His residues and heme cofactors are displayed as sticks, and water molecules as represented as red spheres.
**Figure S8**. Structural arrangement of the tetra‐cysteine site. The major conformers are depicted in the upper panel right (conformer A), while the minor conformers are in the upper panel left (conformer B). The occupancy of the cysteines C_α_, C_𝛽_ and S, and the electron‐density map (2F_0_F_c_, contoured at 1𝜎) around the Cys16 are also shown.
**Figure S9**. Electrostatic surface potential of the tetramer. Electrostatic potential calculated using APBS is mapped onto the molecular surface of the tetramer. Positive and negative potentials are shown in blue and red, respectively, with the electrostatic scale ranging from −5 to +5 kT/e. (A) Lateral and (B) Axial views highlight the distribution of charged patches across the coil and the electrostatic environment surrounding the two heme cofactors.
**Figure S10**. Electrostatic surface potential of the crystal lattice highlighting solvent channels. Electrostatic potential calculated using APBS is mapped onto the molecular surface of the tetramer and neighboring symmetry‐related molecules (over 20 Å) in the crystal lattice. Positive and negative potentials are shown in blue and red, respectively (−5 to +5 kT/e). The packing arrangement generates solvent‐accessible channels lined by alternating charged patches. (A) Crystal lattice over 20 Å highlighting the channels. (B) Axial view of the electrostatic potential mapped onto the crystal lattice. (C) Lateral view of the electrostatic potential mapped onto the crystal lattice.
**Figure S11**. Interactions surrounding the heme cofactors. The propionate groups interactions with nearby water molecules and residues for heme1 (left panel) and heme 2 (right panel) are depicted. The 𝜋‐stacking interactions between Trp2 indole ring and the porphyrin macrocycle is also evidenced, along with their average distance.
**Figure S12**. Superposition comparing the core packing of the crystal structure of apo‐GCSR‐L16CL23C (PDB 7N2Y) and AlphaFold‐generated model of apo‐GRW‐L16CL30H. (Top) Coiled coil core layers. The ribbon backbone of the crystal structure of apo‐GCSR‐L16CL23C is shown in cyan, while the AlphaFold model of apo‐GRW‐L16CL30H is shown in green. Side chains of the *a* (blue) and *d* (red) positions, as well as salt bridges are displayed. Layer number, counted from the N‐ to C‐terminus, are indicated. (Bottom) Geometry of individual hydrophobic layers. Each layer contains the same residue in both apo‐GCSR‐L16CL23C and apo‐GRW‐L16CL30H, except for layer 7 and 9.
**Figure S13**. Helical wheel diagram for apo‐GRW‐L16CL30H. (A) The peptide helices extend from the N‐terminus (emerging from the page) to the C‐terminus (projecting into the page). Interhelical salt bridges (dotted red lines) across Glu (*e*) and Lys (*g*) positions are shown. The hydrophobic core is formed by residues at positions *a* and *d*. Each layer is formed by interactions among three side chains of *a*‐*a′*‐*a″* or *d*‐*d′*‐*d″*. The core interaction for the second heptad is exemplified: Leu9‐Leu9‐Leu9 (dotted purple line) and Leu12‐Leu12‐Leu12 (dotted green line). (B) Cross‐sectional view of the second heptad layer in the apo‐GRW‐L16CL30H 3SCC model. The model was generated from the crystal structure of PDB 7N2Y (Carugo, 2024), with residues in the heptad repeat mutated to match the sequence of GRW‐L16CL30H.
**Figure S14**. Helical wheel diagram for antiparallel 4SCC GRW‐L16CL30H. (A) Two peptide helices (light orange) extend from the N‐terminus (emerging from the page) to the C‐terminus (projecting into the page), while the other two (light purple) extend from the C‐terminus (emerging from the page) to the N‐terminus (projecting into the page) to form an ABAB arrangement. Each hydrophobic layer is formed by interactions among four hydrophobic side chains of *a*‐*d*‐*a′‐d′* or *d‐a‐d′‐a′*. The core interaction for the second heptad (from the N‐terminal) is exemplified: Leu9‐Leu33‐Leu9‐Leu33 (dotted orange line) and Leu12‐His30‐Leu12‐His30 (dotted brown line). Considering a canonical antiparallel 4SCC with an *a/d* hydrophobic core, no interhelical salt bridges are allowed to be formed due to charge repulsion between *e*–*e′* and *g‐g′*positions. (B) Cross‐sectional view of the second heptad layer in the GRW‐L16CL30H 4SCC model. The model was generated from the crystal structure of PDB 8A3G (Polizzi et al., 2017), with residues in the heptad repeat mutated to match the sequence of GRW‐L16CL30H.

## Data Availability

The data that support the findings of this study are available from the corresponding author upon reasonable request.
