# Supplementary material for: The X‐ray crystallographic structure of a designed, self‐assembling, and heme‐binding protein
Source: Protein Sci. 2026 Oct 27;35(11):e70829. doi: 10.1002/pro.70829 (PMC13711483; doi:10.1002/pro.70829)
Supplement: Supplementary file 1 [file PRO-35-e70829-s001.pdf]

Supporting Information

The X-Ray crystallographic structure of a designed, self-assembling and heme-binding protein

Iago de Assis Modenez [1,2], Véronique Roig-Zamboni [3], Philippe Leone [4*],
Anabella Ivancich [2*], Vincent L. Pecoraro [1*]

[1] *Department of Chemistry, University of Michigan, Ann Arbor, MI, United States.*
[2] *Bioénergétique et Ingénierie des Protéines, Unité Mixte de Recherche UMR 7281, CNRS and Aix-Marseille Université, Marseille, France.*
[3] *Architecture et Fonction des Macromolécules Biologiques, CNRS and Aix-Marseille Université, Marseille, France.*
[4] *Ingénierie des Systèmes Macromoléculaires (UMR 7255), Institut de Microbiologie de la Méditerranée, CNRS and Aix-Marseille Université, Marseille, France.*

[*] Corresponding authors
E-mail: pleone@imm.cnrs.fr, aivancich@imm.cnrs.fr, vlpec@umich.edu

Table S1. Interactions between the tetramer and neighboring chains interconnecting the tetramers to generate the three-dimensional crystal lattice (4 Å-cutoff)

Chain	Main tetramer	Chain	Neighboring tetramer	Distance (Å)	Interaction type
C	Glu28	A	Lys31	3.91	Salt bridge
A	Lys24	B	Glu34	2.99<br>3.40	Salt bridge
A	Lys29	B	Glu35	2.88<br>3.49	Salt bridge
B/D	heme2	A	Lys22	3.78	H-bond
B/D	heme2	A	Lys29	2.74	H-bond
D	Lys3	C	Glu34	3.82	Salt bridge
D	Lys10	C	Glu35	3.25	Salt bridge
A	Lys3	C	Glu35	3.26<br>3.38	Salt bridge

Table S2. Side chain rotamers (χ_1 and χ_2) for residues at *a* and *d* position

Chain A	χ_1	χ_2	Chain B	χ_1	χ_2	Chain C	χ_1	χ_2	Chain D	χ_1	χ_2
**Trp2**	-64.1	109.2		-71.7	107		-71.8	107.8		-56.6	100.3
**Leu5**	-70.1	175.7		-70.3	175.7		-72.6	178.2		-68.8	173.4
**Leu9**	-81.8	159.2		-150.8	69.3		-165.8	67.4		-79.5	162.7
**Leu12**	108.8	-176.5		-159.8	149		-103.3	-169.6		-161.5	33.6
**Cys16**	-73.3	-		-62.7	-		-67	-		-75.8	-
**Leu19**	-169.5	48.2		-178.9	60.2		-166.9	58.6		-169.3	50.5
**Leu23**	-72.8	165.9		-72.5	168.4		179.4	73.2		-70.5	167.2
**Leu26**	-67.1	177.2		-76.4	169.3		-76.1	169.3		-77	172.6
**His30**	-83.8	71.5		-82.3	76.2		-81.2	74		-85.1	62.3
**Leu33**	-70.5	155.3		-64.7	170.7		-49.2	-178.9		-96.5	61.8

Table S3. Average buried surface area by heptad position for the core layers (4-9), termini ends (layer 1-2 and 11-12) and the whole tetramer (layers 1-12)

Position	Core layers (4-9) (%)	Termini layers (1-2 and 11-12) (%)	Total (layers 1-12) (%)
**a**	93	48	75
**b**	3	2	2
**c**	26	25	22
**d**	83	21	66
**e**	57	38	46
**f**	0	0	0
**g**	54	27	39

Table S4. Coiled coil parameters

α**-helix parameter**	**Chain A**	**Chain B**	**Chain C**	**Chain D**	**Average**
α-helix radius (Å)	2.35	2.33	2.35	2.32	2.34 ± 0.02
Angular frequency (°/residue)	99.5	95.9	95.3	98.6	97.3 ± 2.1
Rise per residue (Å)	1.50	1.45	1.48	1.52	1.49 ± 0.03
Residues per α-helical turn	3.6	3.7	3.8	3.7	3.7 ± 0.08
**superhelix parameter**					
Supercoil radius (Å)	-	-	-	-	7.0
Tilt angle relative to superhelical axis (°)	-	-	-	-	13.3
Supercoil pitch (Å)	-	-	-	-	186
Translation of the supercoiled helix along the superhelical axis (Å/residue)	1.45	1.40	1.43	1.43	1.44 ± 0.03
Residues per superhelix turn (residues/turn)	124	129	126	127	127 ± 2

Table S5. Sequence of relevant peptides discussed in this study

**PEPTIDE**	**1**	**2**	**9**	**16**	**23**	**30**	**37**
**GRAND (GR)**	Ac-G	LKALEEK	LKALEEK	LKALEEK	LKALEEK	LKALEEK	G-NH_2
**GR-L2W (GRW)**	Ac-G	**W**KALEEK	LKALEEK	LKALEEK	LKALEEK	LKALEEK	G-NH_2
**GRW-L16CL30H**	Ac-G	**W**KALEEK	LKALEEK	**C**KALEEK	LKALEEK	**H**KALEEK	G-NH_2
**GRAND-CoilSer (GRCS)**	Ac-E	WEALEKK	LAALESK	LQALEKK	LQALEKK	LEALEH	G-NH_2
**GRCS-L16CL23C**	Ac-E	WEALEKK	LAALESK	**C**QALEKK	**C**QALEKK	LEALEH	G-NH_2

Crystallization conditions used for the GRW-L16CL30H scaffold in complex with heme.

As described in the Materials and Methods section, crystals that were suitable for resolving the structure were obtained at high protein concentrations (50 mg/mL of monomeric peptide) and using mostly acidic crystallization solutions/buffers. **Fig. S1a** illustrates different conditions used and samples, thus the one resulting in the crystals used to resolve the structure reported here (sample named “S11”), which was obtained using a crystallization solution of 2M NaCl + 2M AmSO4 (condition H1 (position 85)) and the sample named “S12b”, for which trials were based on conditions of S11.

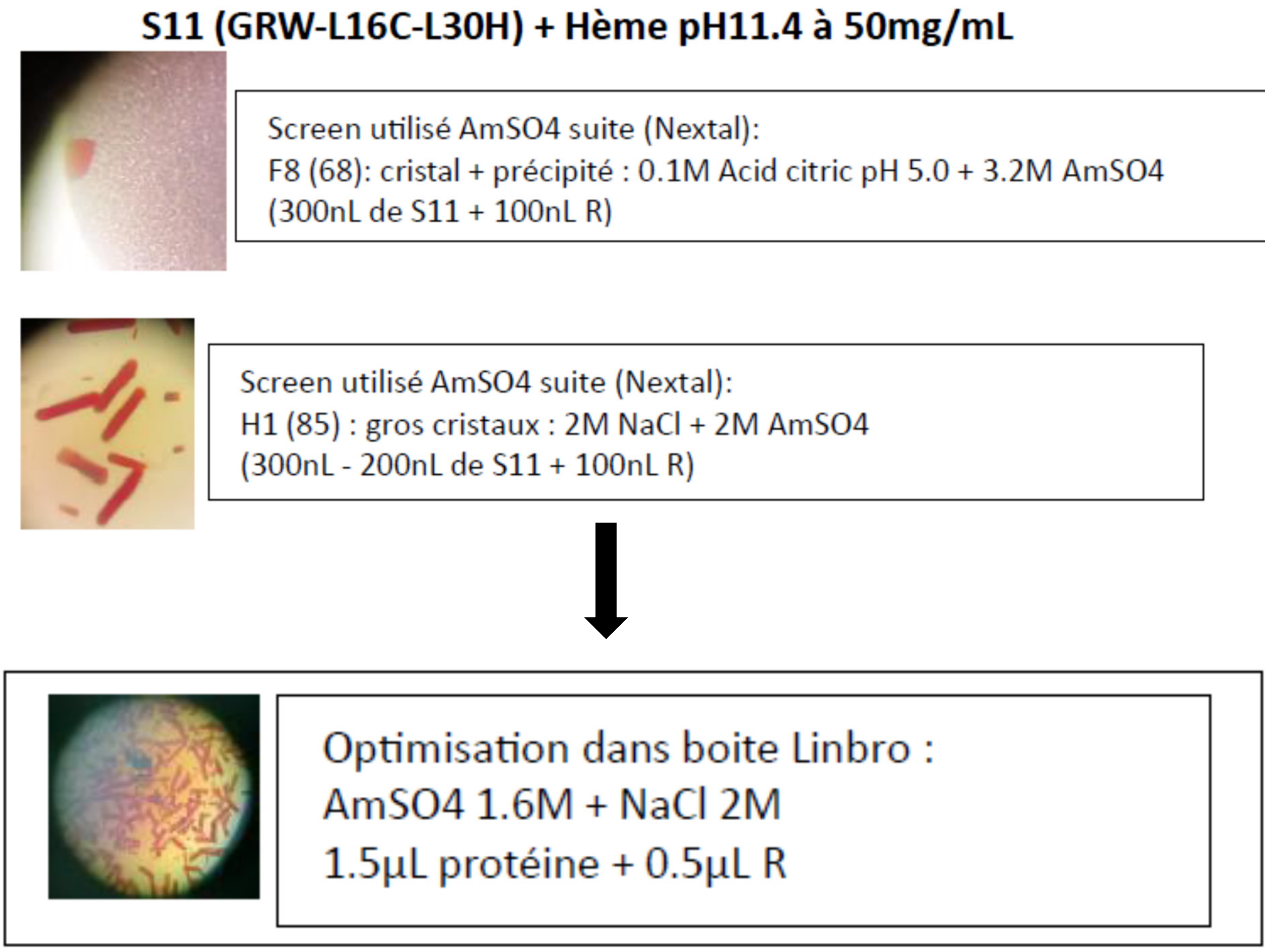

S12b GRW-L16C-L30H + Hème pH11.34 à 50mg/mL

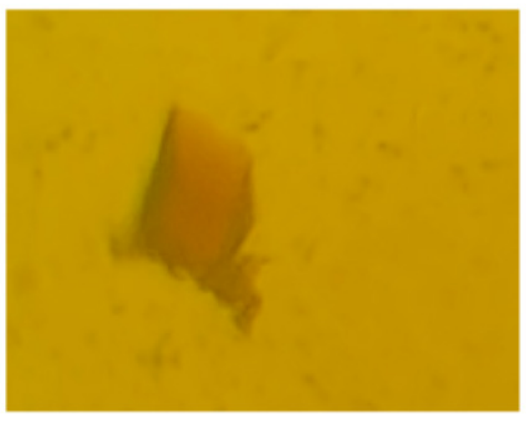

Screen utilisé AmSO4 suite (Nextal):
F7 (67): 0.1M Acid citric pH4.0 + 3.2M AmSO4
(200nL p + 100nL

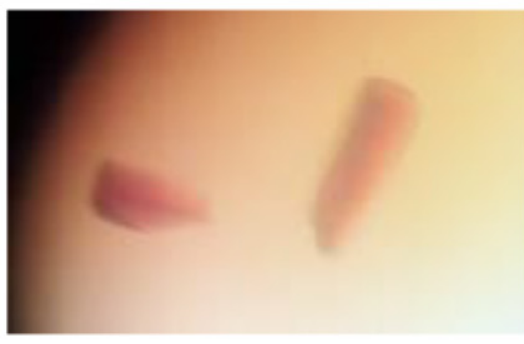

F7 (67): 0.1M Acid citric pH4.0 + 3.2M AmSO4
(300nL p + 100nL R)

8	S12b<br>Linbro1 A2	25% glycerol
9	S12b<br>Linbro1 A2	25% glycerol
10	S12b<br>Linbro1 A2	25% glycerol

Beamline Proxima 2 du 23/03/24

Figure S1a. Crystals of holo-GRW-L16CL30H obtained using different crystallization conditions.

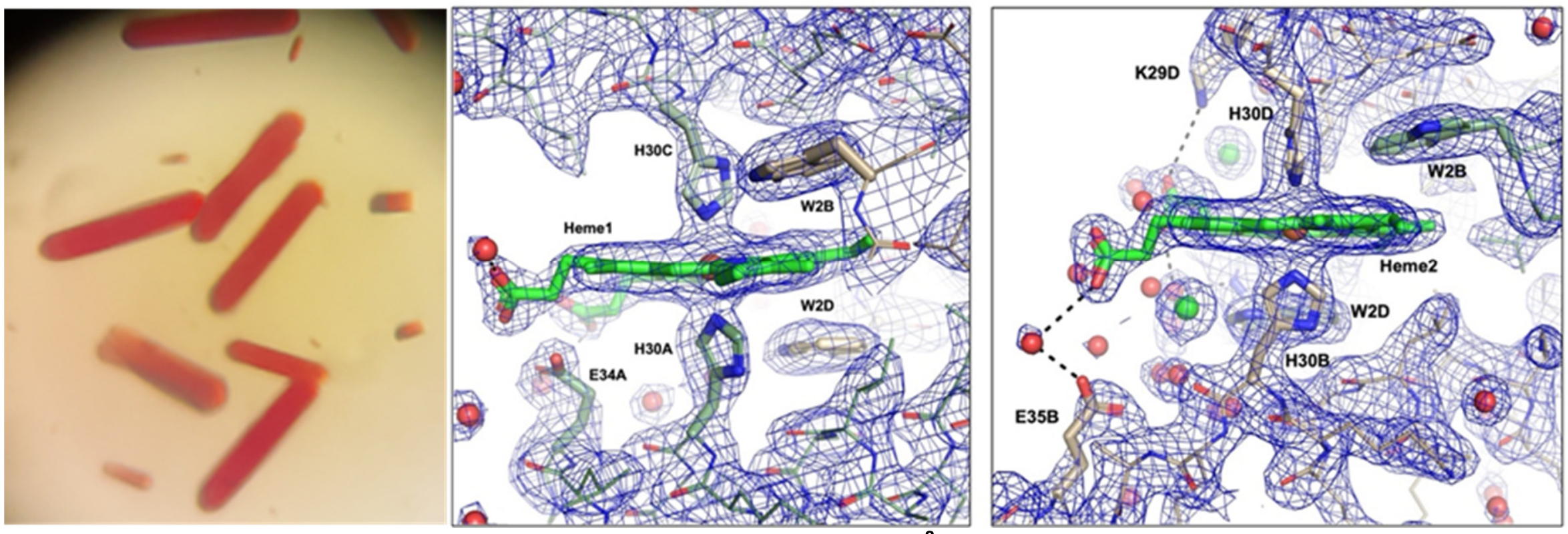

Figure S1b. Crystals of holo-GRW-L16CL30H diffracted up to 1.9 Å resolution. Optical image of crystals of holo-GRW-L16CL30H **(Left)**. Representative electron-density map ($2F_0F_c$, contoured at 1σ) around the heme1 **(Middle)** and heme2 **(Right).**

Crystals packing

Heme-heme contacts between symmetry-related tetramers contribute to the crystal packing, generating an extended supramolecular lattice (Fig. S2). Although the Fe-Fe separation between facing hemes is 9.3 Å, reflecting a markedly offset stacking geometry, the shortest intermolecular heme-heme contact (～3.7 Å) occurs between a vinyl carbon of one heme and a carbon atom of the imidazole ring of a heme in a neighboring tetramer. The propionate substituents of each heme adopt opposite orientations, rotated by approximately 180°, projecting away from the heme-heme interface and thereby minimizing steric interference at the packing interface. The heme-heme contacts occur exclusively between tetramers located laterally in the lattice, giving rise to linear assemblies that propagate across the crystal unit. In addition to these interactions, several salt bridges (Glu→Lys) and hydrogen-bonding interactions contribute to the packing (see Fig. S3 and Table S1).

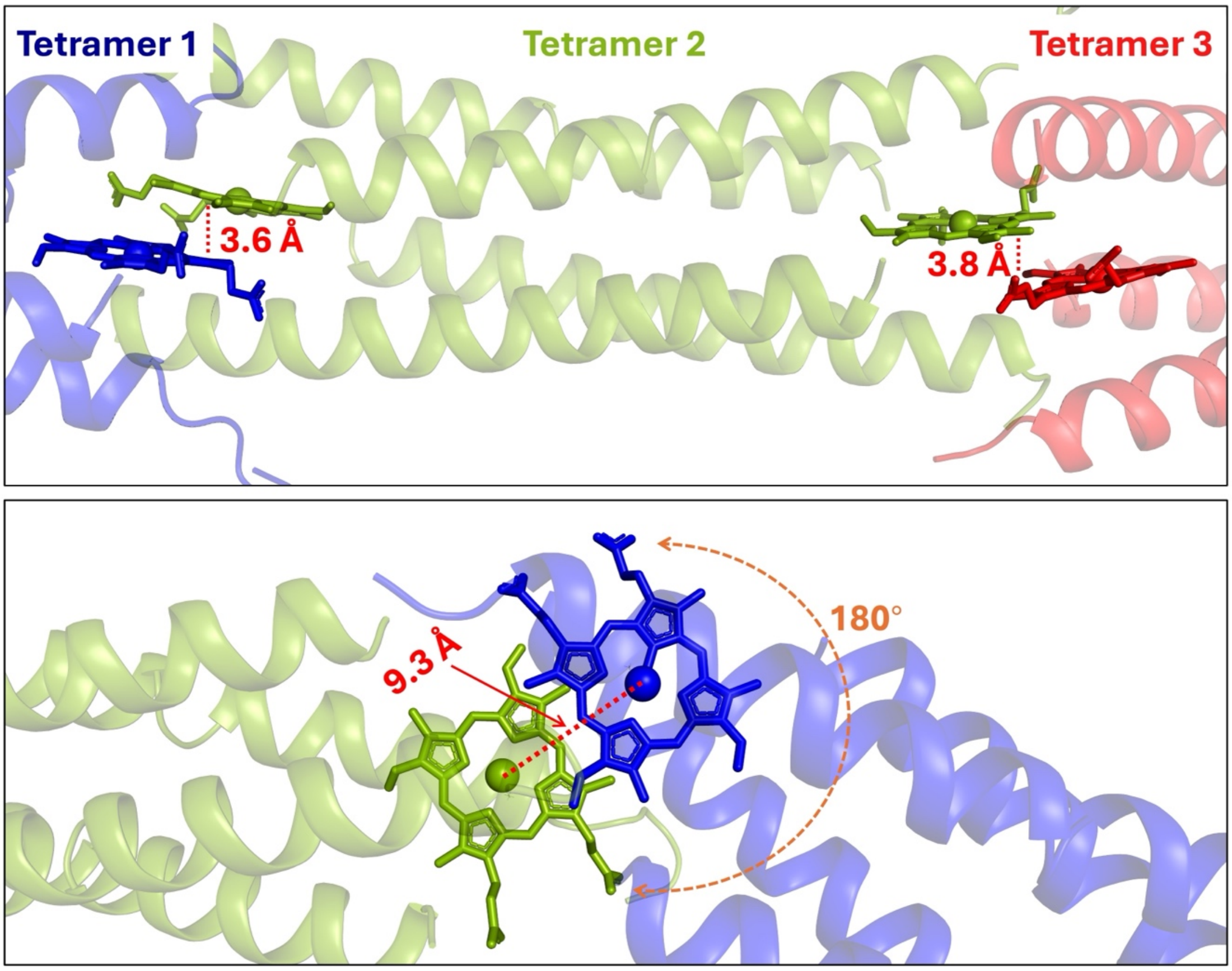

Figure S2. Porphyrin-porphyrin contacts between symmetry-related tetramers in the crystal lattice. (Top) Closest intermolecular contact between neighboring heme cofactors, occurring between a vinyl carbon of one heme and a carbon atom of the imidazole ring from a neighboring tetramer. **(Bottom)** Top view of two symmetry-related tetramers highlighting the offset arrangement of the heme cofactors between the tetramers.

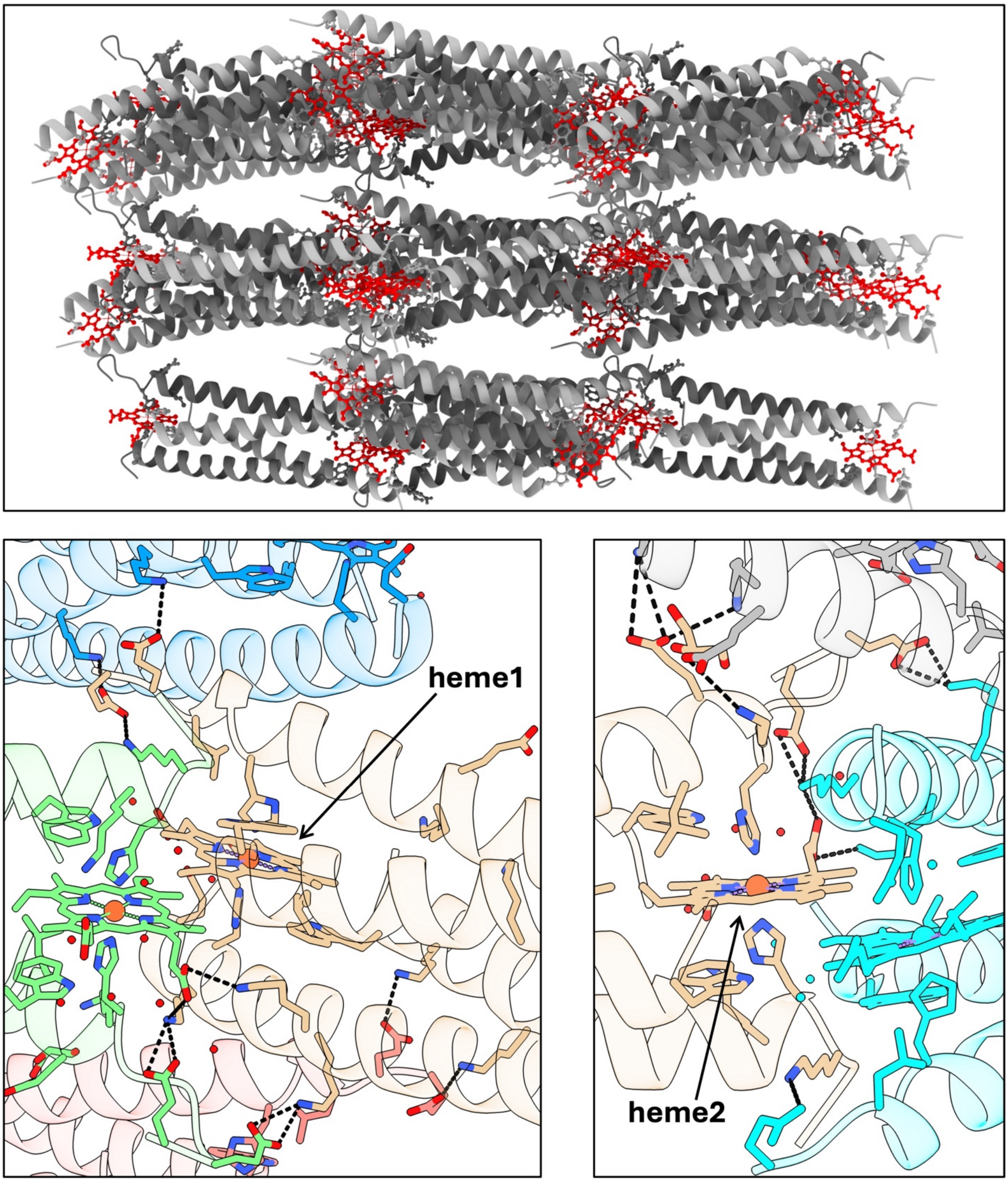

Figure S3. Heme-heme and salt bridge contacts between symmetry-related tetramers. (Top) Heme-heme contacts generate an approximately linear arrangement of tetramers across the crystal lattice. **(Bottom)** Salt-bridges between Glu and Lys residues connect Tetramer 1 (light brown) with neighboring tetramers. In the left panel, heme1 is shown, highlighting salt bridges between this tetramer and three neighboring tetramers (blue, light green, and salmon). In the right panel, heme 2 is shown, where several salt bridges connect this tetramer with two neighboring tetramers (light blue and light gray).

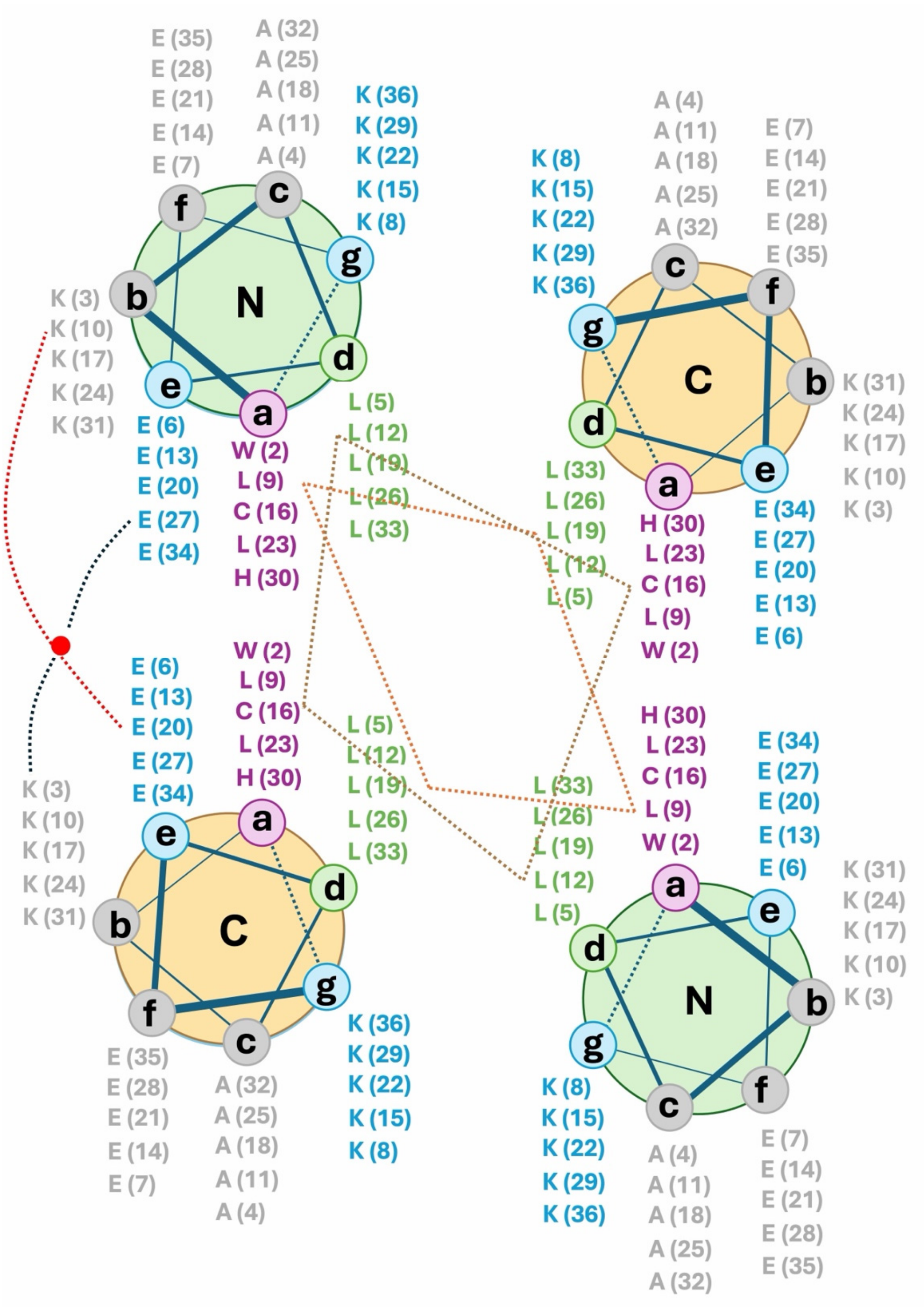

Figure S4. Helical wheel diagram for antiparallel holo-GRW-L16CL30H. Two peptide helices (light green) extend from the N-terminus (emerging from the page) to the C-terminus (projecting into the page), while the other two (light yellow) extend from the C-terminus (emerging from the page) to the N-terminus (projecting into the page) to form an out-of-register ABAB arrangement. Each hydrophobic layer is formed by interactions among four hydrophobic side chains of *a-d-a′-d′* or *d-a-d′-a′*. The core interaction for the 2nd heptad (from the N-terminal) is exemplified: Leu9-Leu19-Leu9-Leu19 (dotted orange line) and Leu12-Cys16-Leu12-Cys16 (dotted brown line). The interhelical salt bridge between K10 and E20 is shown as a dotted red trace, while the water-mediated (red sphere) hydrogen bond between K3 and E27 is shown as dotted black traces.

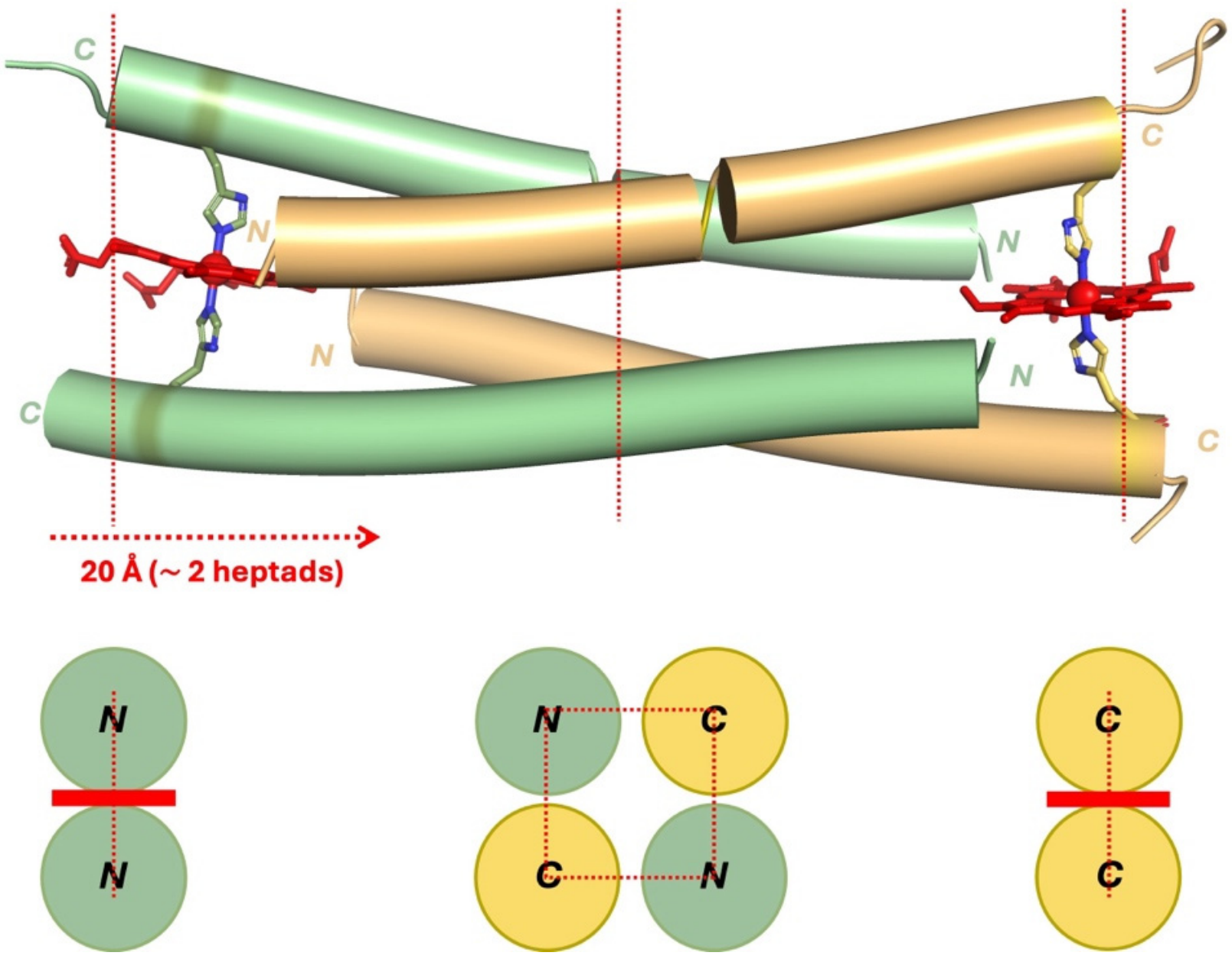

Figure S5. Cylinder representation of holo-GRW-L16CL30H. Cross-sections of the helical arrangements are shown for the center and ends of the coils.

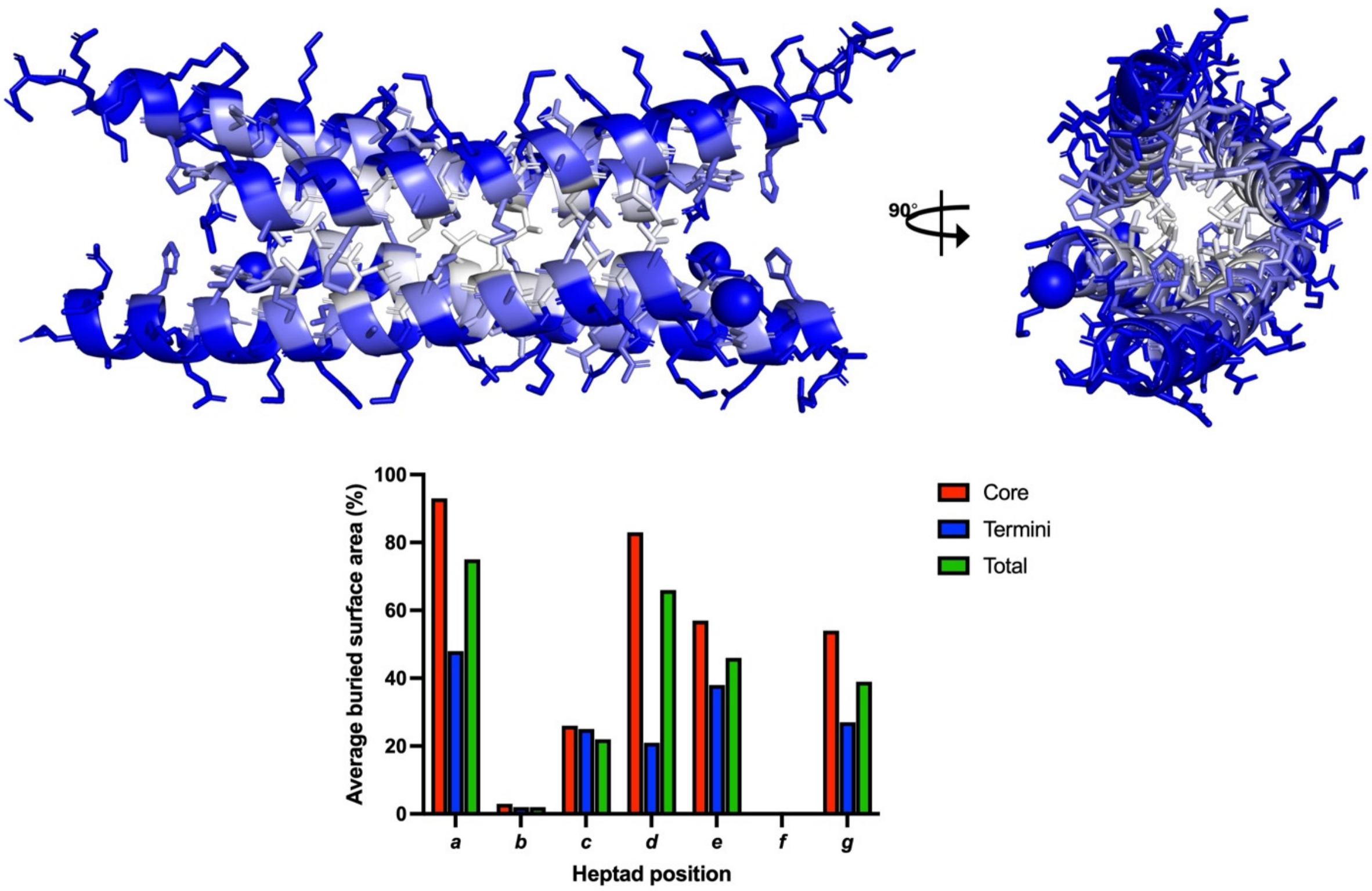

Figure S6. Solvent exposure mapped onto the tetramer structure. (Top) Average residue solvent exposure calculated using ArealMol from CCP4 suite and mapped onto the structure. The protein is colored according to normalized solvent accessibility, ranging from white (least exposed, b = 0) to dark blue (most exposed, b = 1). **(Bottom)** Average buried surface area (%) bar chart per heptad position for tetramer core, termini, and total.

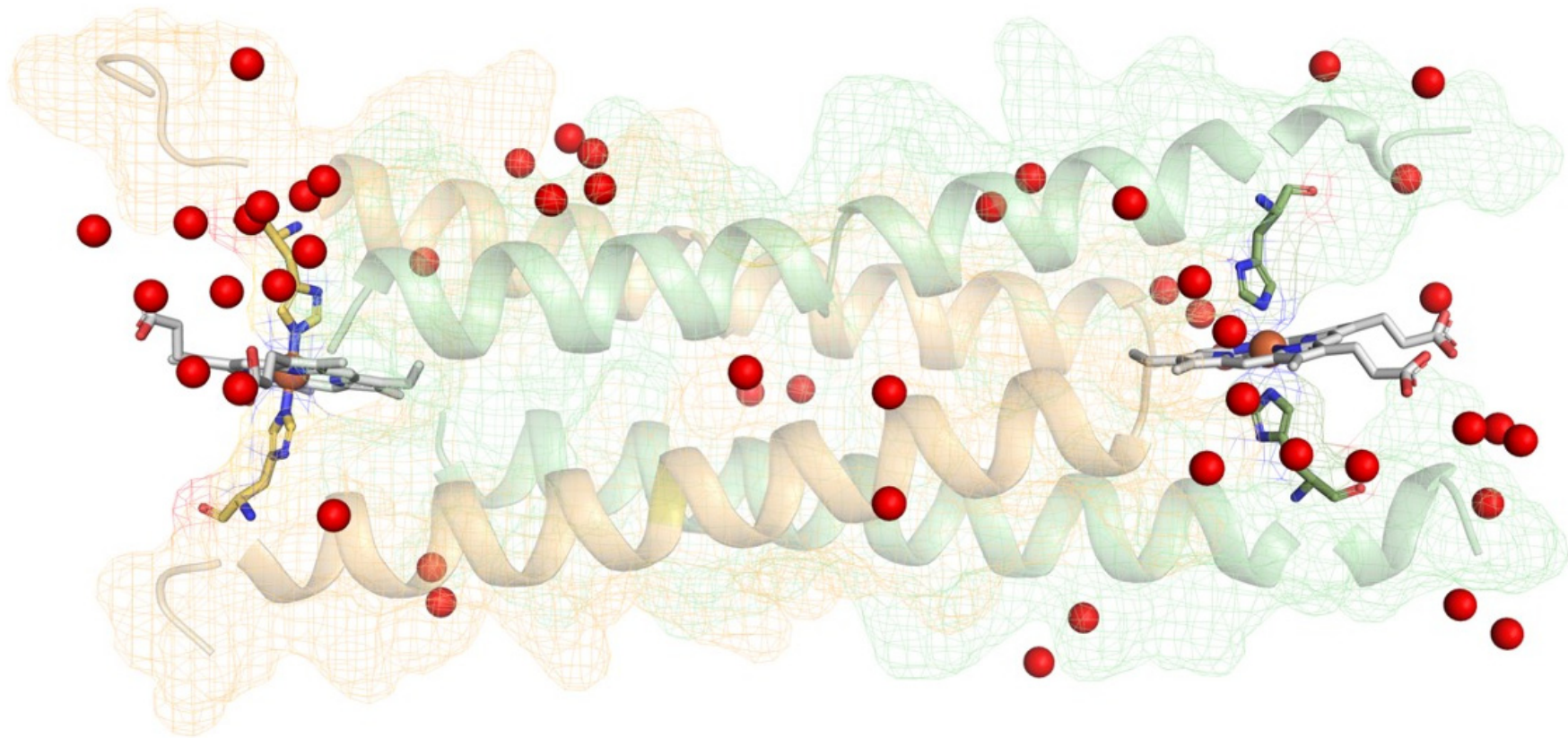

Figure S7. Water accessibility to the tetramer. A mesh surface surrounding the tetramer highlights regions of increased hydration near the heme cofactors. Most of the heme macrocycle remains solvent-exposed. Protein backbones are shown as ribbon, the coordinating His residues and heme cofactors are displayed as sticks, and water molecules as represented as red spheres.

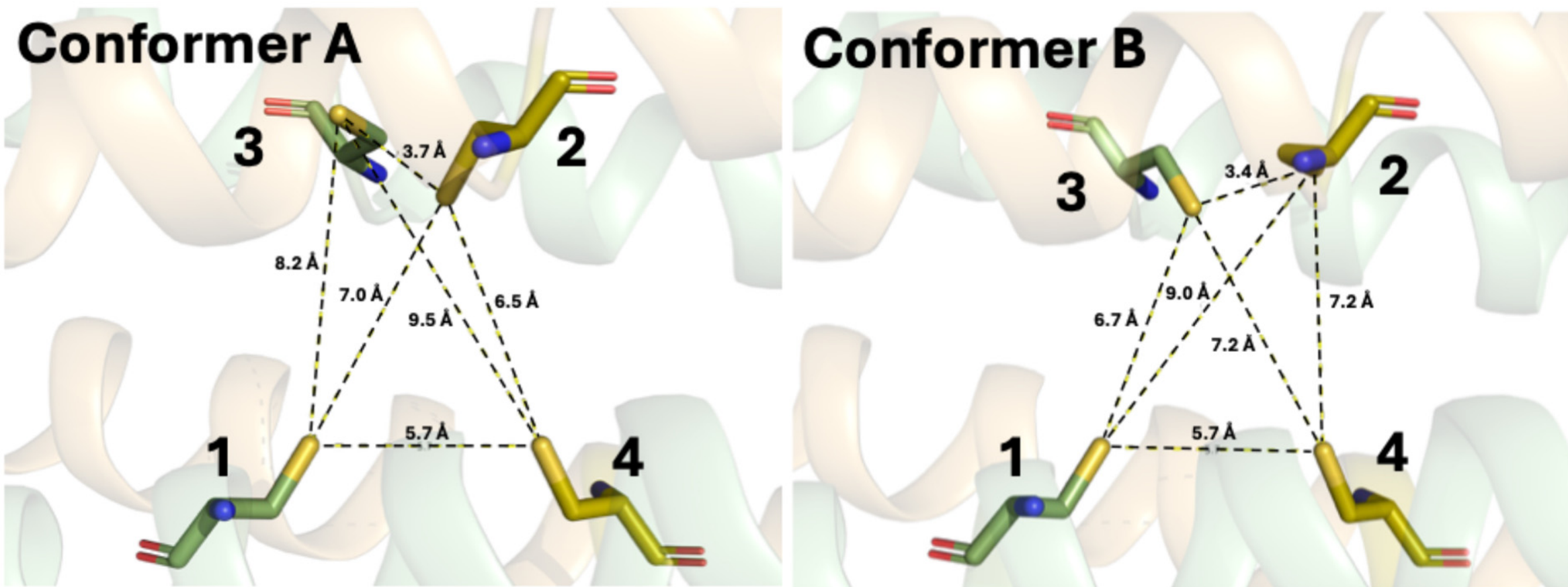

Cys	1 (chain A)	2 (chain B)	3 (chain C)	4 (chain D)
Cα	1.0	A 0.63<br>B 0.37	A 0.54<br>B 0.46	1.0
Cβ	1.0	A 0.63<br>B 0.37	A 0.54<br>B 0.46	1.0
S	1.0	A 0.63<br>B 0.37	A 0.54<br>B 0.46	1.0

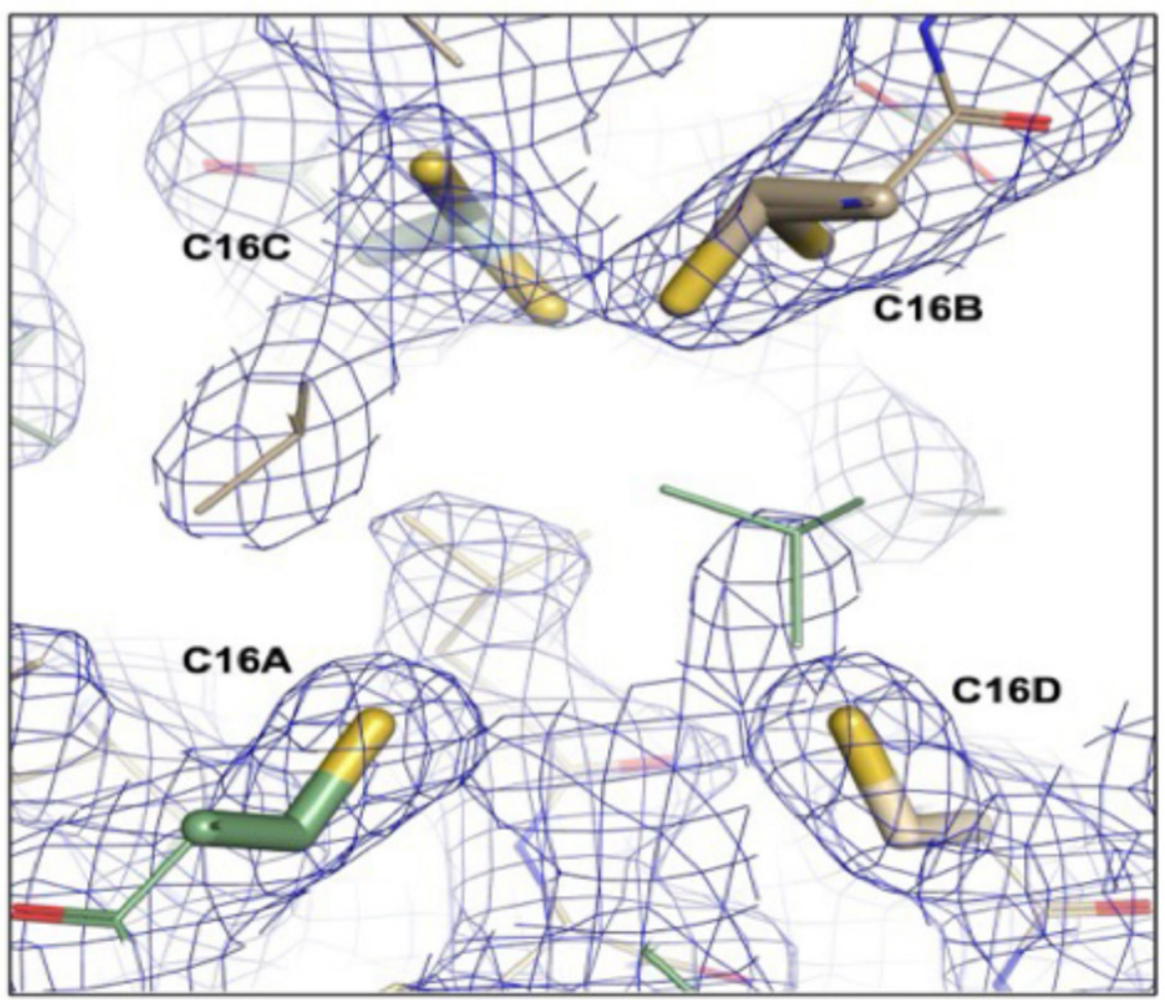

Figure S8. Structural arrangement of the tetra-cysteine site. The major conformers are depicted in the upper panel right (conformer A), while the minor conformers are in the upper panel left (conformer B). The occupancy of the cysteines Cα, Cβ and S, and the electron-density map ($2F_0F_c$, contoured at 1σ) around the Cys16 are also shown.

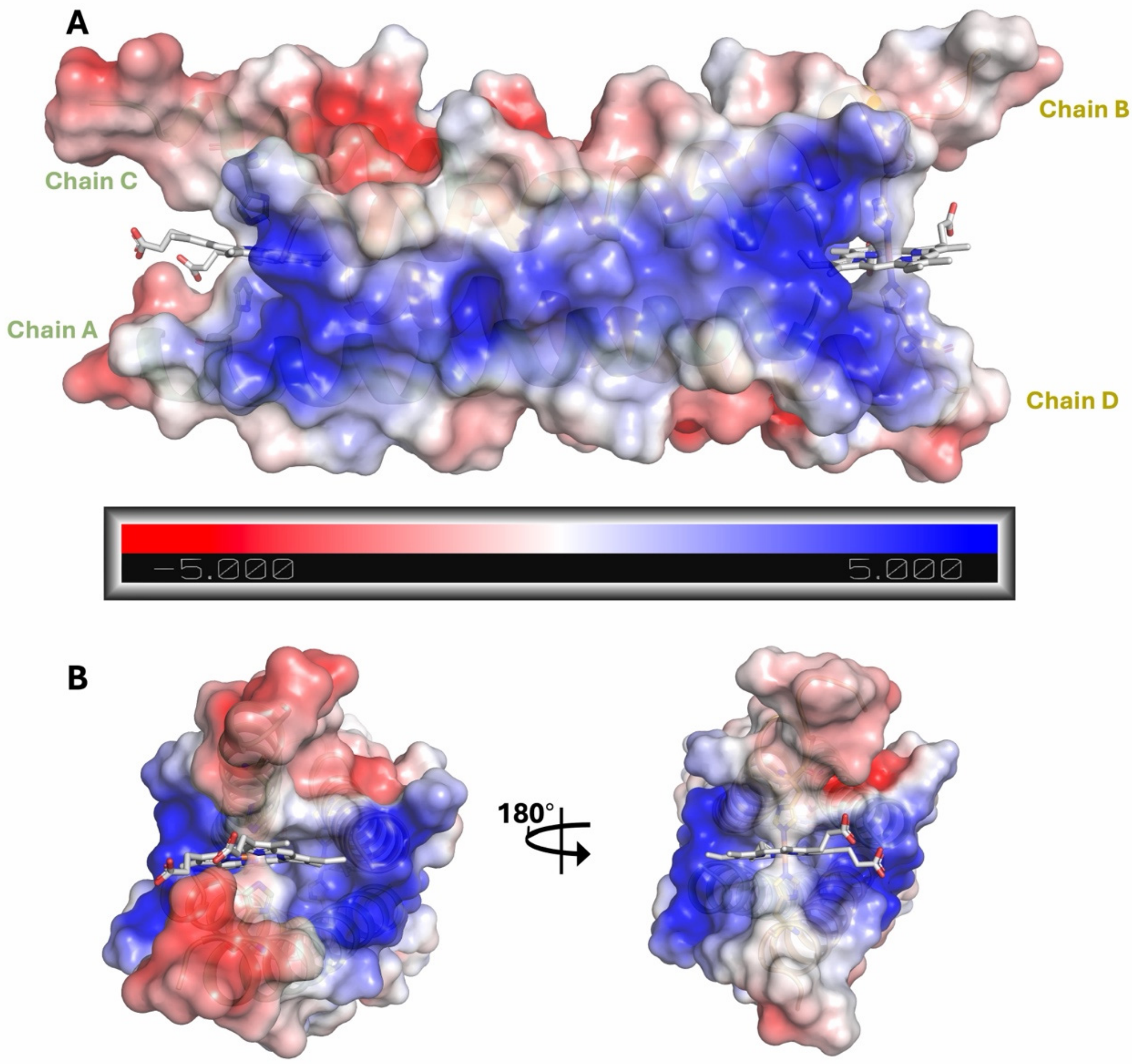

Figure S9. Electrostatic surface potential of the tetramer. Electrostatic potential calculated using APBS is mapped onto the molecular surface of the tetramer. Positive and negative potentials are shown in blue and red, respectively, with the electrostatic scale ranging from -5 to +5 kT/e. **A.** Lateral and **B.** Axial views highlight the distribution of charged patches across the coil and the electrostatic environment surrounding the two heme cofactors.

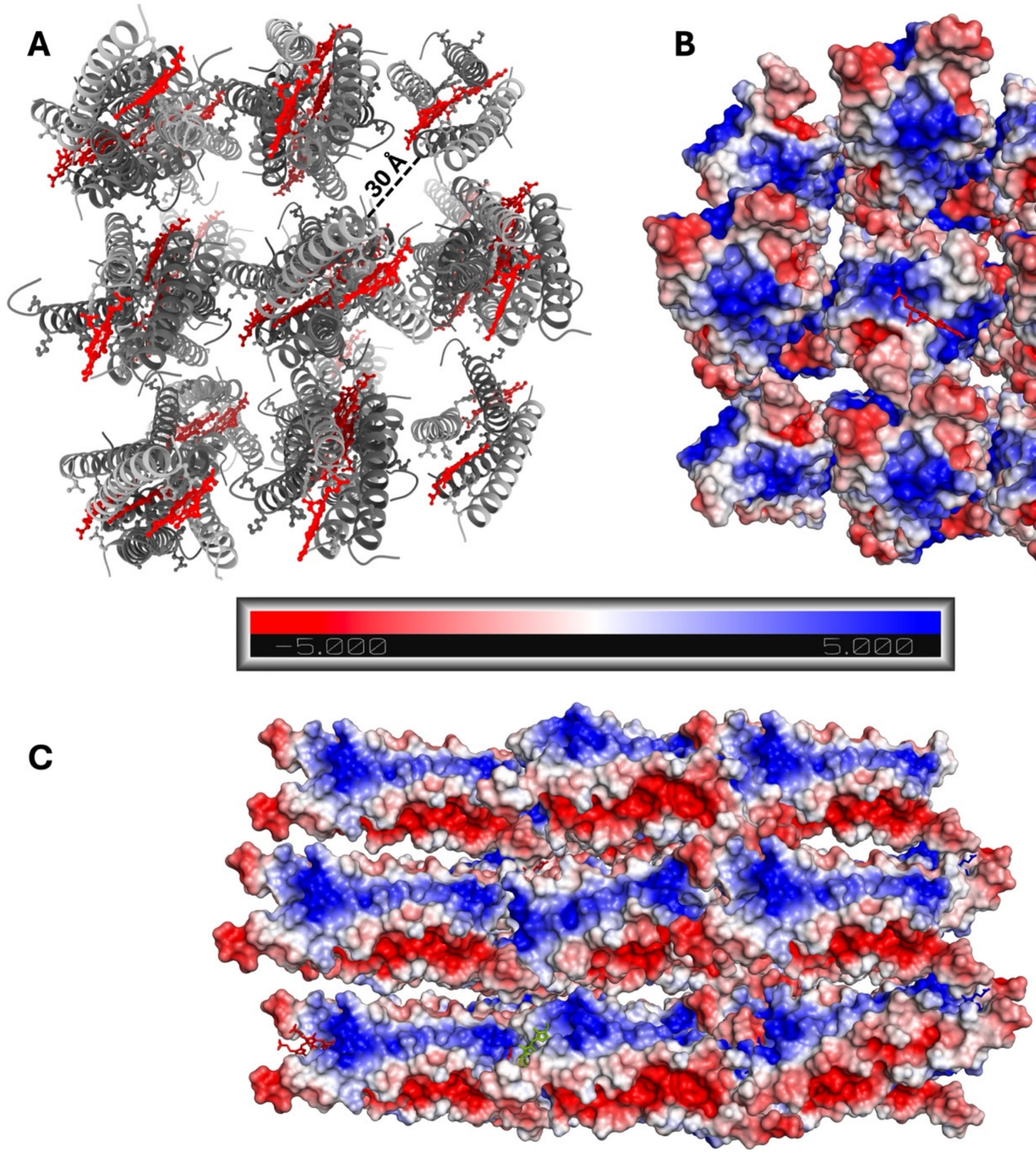

Figure S10. Electrostatic surface potential of the crystal lattice highlighting solvent channels. Electrostatic potential calculated using APBS is mapped onto the molecular surface of the tetramer and neighboring symmetry-related molecules (over 20 Å) in the crystal lattice. Positive and negative potentials are shown in blue and red, respectively (-5 to +5 kT/e). The packing arrangement generates solvent-accessible channels lined by alternating charged patches. **A.** Crystal lattice over 20 Å highlighting the channels. **B.** Axial view of the electrostatic potential mapped onto the crystal lattice. **C.** Lateral view of the electrostatic potential mapped onto the crystal lattice.

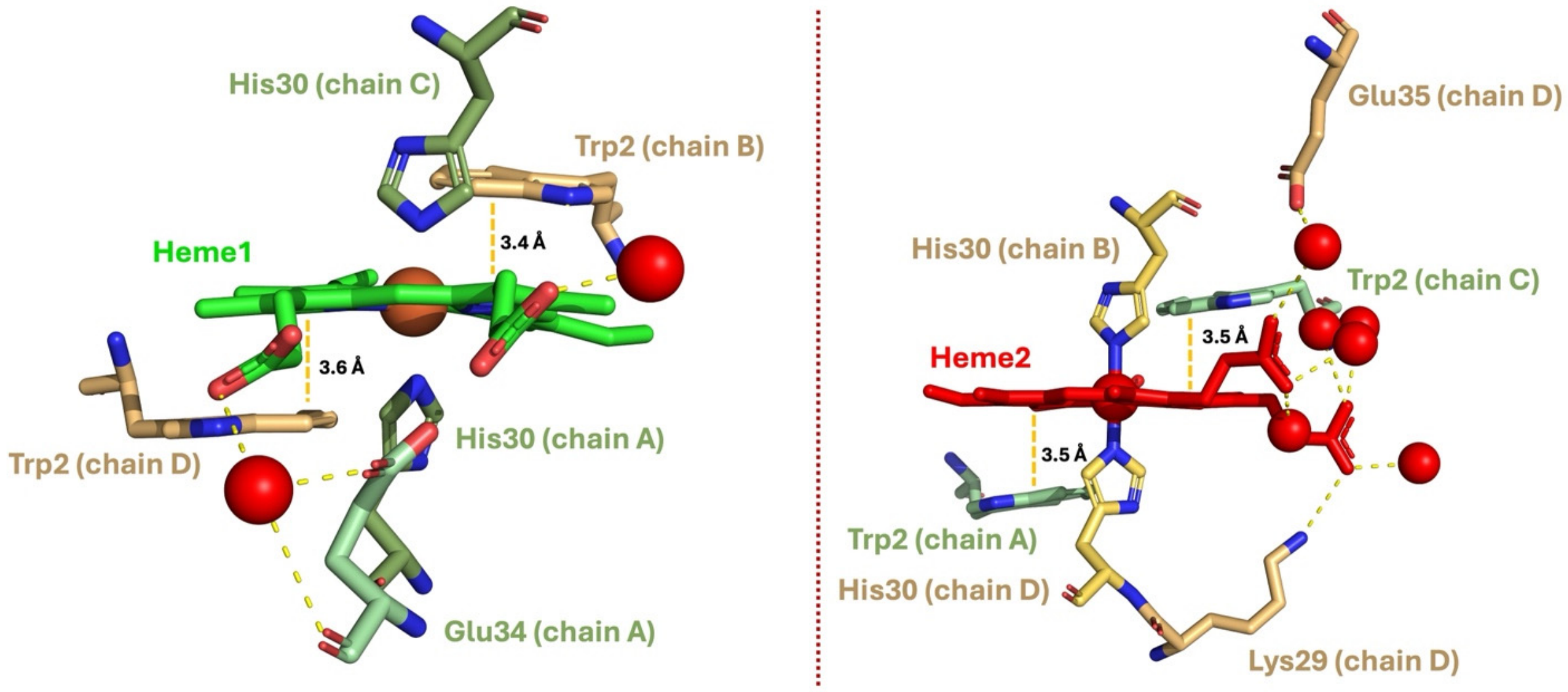

Figure S11. Interactions surrounding the heme cofactors. The propionate groups interactions with nearby water molecules and residues for heme1 (left panel) and heme 2 (right panel) are depicted. The π-stacking interactions between Trp2 indole ring and the porphyrin macrocycle is also evidenced, along with their average distance.

Structural features of parallel apo-GRW-L16CL30H inferred from related GRAND-CoilSer analogues

Previous works from our group introduced a family of *de novo* designed peptides known as GRAND (GR)[1] (Table S5), which self-assemble into 3SCCs in their apo forms at pH ≥ 5. These peptides are based on a classical heptad repeat motif (Leu^{a}Lys^{b}Ala^{c}Leu^{d}Glu^{e}Glu^{f}Lys^{g}), in which hydrophobic residues at positions *a* and *d* drive oligomerization into a well-folded, parallel, and symmetric trimer via hydrophobic core packing. Additional stabilization is provided by interhelical electrostatic interactions between Glu (*e*-position) and Lys (*g*-position) residues. Destabilizing interactions associated with the helix dipole moment are minimized by capping the terminal α-NH_3^+ and α-COO^- groups through N-acetylation and C-amidation, respectively. The parental GR sequence can be systematically modified to introduce a Trp residue at position 2, enabling accurate spectroscopic determination of peptide concentration, as well as coordinating residues at *a* and/or *d* positions of the heptad repeat to create defined metal-binding sites. Spectroscopical studies have shown that these engineered peptides can bind a wide range of heavy metals and metalloids,[2–6] and first-row transition metals,[7–10] within the hydrophobic core of the trimeric assembly. A detailed structural understanding of metal coordination within this peptide family has been achieved from high-quality crystal structures of a closely related variant, GRAND-CoilSer (GRCS, Table S5), which serves as a crystallographic analogue for the broader GR family.[11–13] In this construct, a His residue at the 35th position (within the last heptad) enables the formation of external Zn(II) coordination sites between symmetry-related trimers, thereby promoting three-dimensional crystal packing.[14]

GRW-L16CL30H is a scaffold derived from the parental GRW sequence, in which a Cys is introduced at position 16 (*a*) and a His at position 30 (*a*), respectively [15,16] (Table S5). Although no crystal structure is currently available for the apo form of GRW-L16CL30H, analytical ultracentrifugation studies confirmed that this peptide assembles as a 3SCC at pH ≥ 5. Based on established GRAND design principles and the structural features observed in the related GRAND-CoilSer (GCSR-L16CL23C) crystal structure,[13] we hypothesize that apo-GRW-L16CL30H adopts a parallel 3SCC assembly. The corresponding structural model predicts a length of approximately 53 Å and a diameter of 12 Å, comprising just over ten helical turns (Fig. S12), with coiled coil interactions similar to those observed in crystallized GRCS constructs. In these related structures, the three parallel helices have an average interhelical axis distance of 7.5 Å and bury ~35% of the total monomer surface area upon trimer formation.

In the proposed model, the core at the interface of the three α-helices is predicted to contain over twenty-four hydrophobic residues occupying *a* and *d* positions of the heptad repeat (e.g. Trp2, Leu5, Leu9, Leu12, Leu19, Leu23, Leu26, and Leu33), which engage in canonical knobs-into-holes interactions along the threefold symmetry axis, resulting in eight hydrophobic layers. Each layer is formed by interactions among three apolar side chains of the type *a-a'-a"* or *d-d'-d"* where *a/d* refers to positions on helix I, *a'/d'* to helix II, and *a"/d"* to helix III (Fig. S13). The model also predicts that the Cα-Cβ vectors of all Leu occupying *a* and *d* positions are oriented toward the superhelical axis. Similarly, Cys16 and His30 are predicted to occupy symmetric layers, giving rise to potential Cys-Cys-Cys and His-His-His coordinating layers. Based on the GCSR-L16CL23C structure, the model further predicts that interhelical electrostatic stabilization is provided by recurring salt bridges between Glu residues at the *e* positions (e.g. Glu6, Glu13, etc.) and Lys residues at the *g* positions (e.g. Lys8, Lys15, etc.) of adjacent helices.

Interestingly, for this canonical 3SCC, the AlphaFold 3 prediction shows good agreement with the crystallographic data obtained from the GRCS-L16CL23C analogue (RMSD = 0.39 Å) (Fig. S12). The model contains ten core interactions and twelve interhelical salt bridges. This suggests that the core packing, interhelical interactions, and overall assembly of apo-GRW-L16CL30H are likely comparable to those observed in the apo-GCSR-L16CL23C crystal structure.

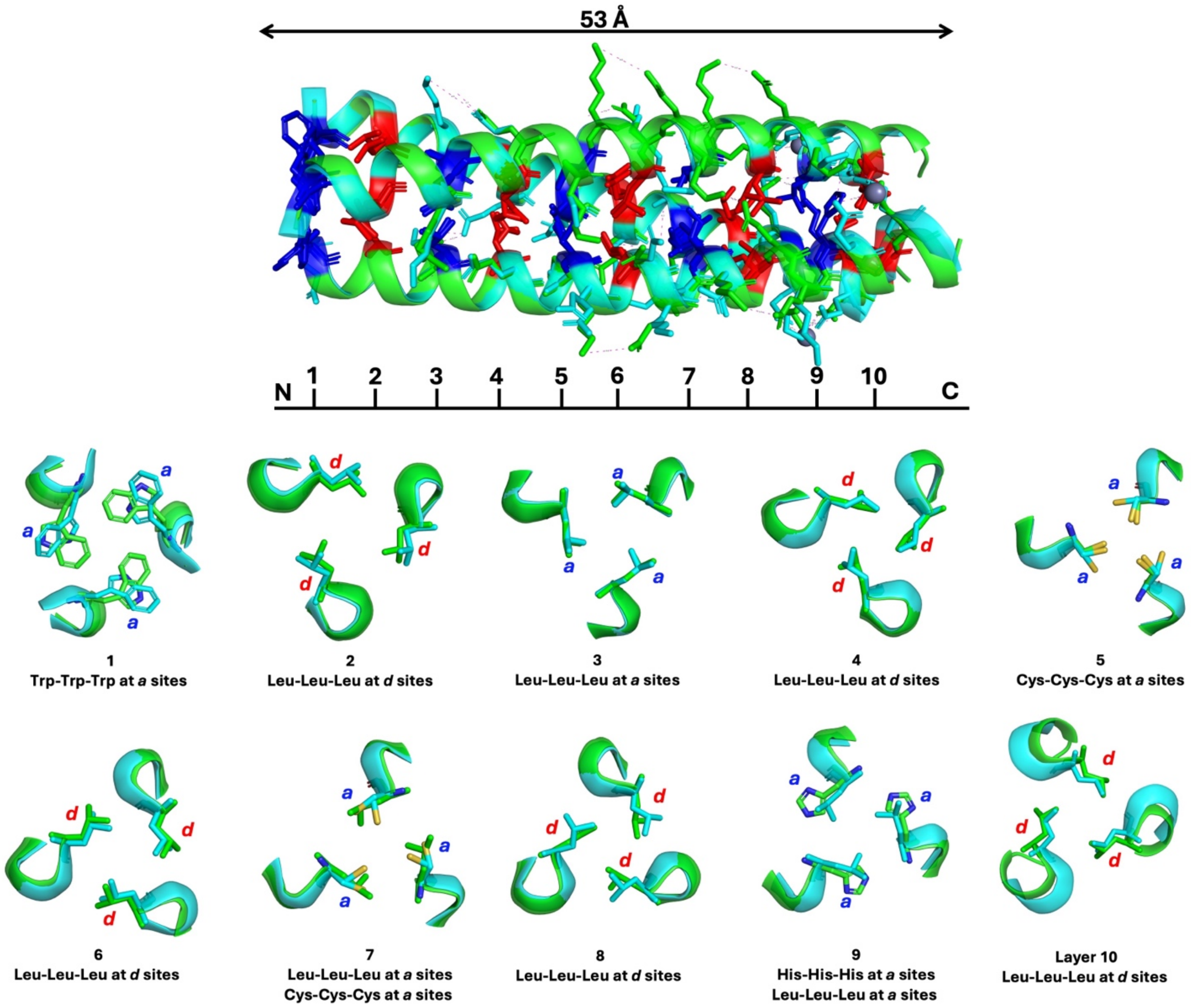

Figure S12. Superposition comparing the core packing of the crystal structure of apo-GCSR-L16CL23C (PDB 7N2Y) and AlphaFold-generated model of apo-GRW-L16CL30H. (Top) Coiled coil core layers. The ribbon backbone of the crystal structure of apo-GCSR-L16CL23C is shown in cyan, while the AlphaFold model of apo-GRW-L16CL30H is shown in green. Side chains of the *a* (blue) and *d* (red) positions, as well as salt bridges are displayed. Layer number, counted from the N- to C-terminus, are indicated. (Bottom) Geometry of individual hydrophobic layers. Each layer contains the same residue in both apo-GCSR-L16CL23C and apo-GRW-L16CL30H, except for layer 7 and 9.

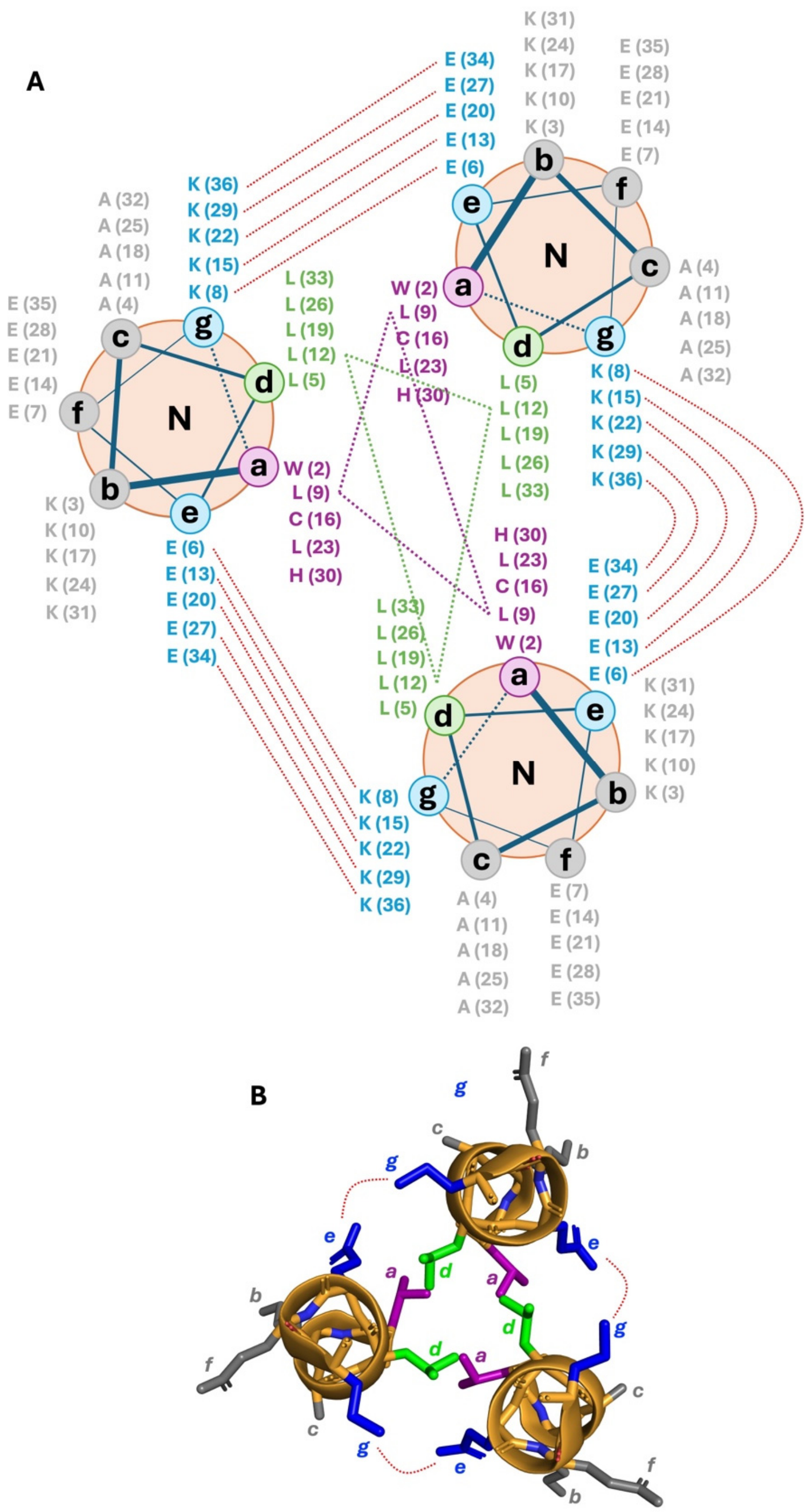

Figure S13. Helical wheel diagram for apo-GRW-L16CL30H. A. The peptide helices extend from the N-terminus (emerging from the page) to the C-terminus (projecting into the page). Interhelical salt bridges (dotted red lines) across Glu (*e*) and Lys (*g*) positions are shown. The hydrophobic core is formed by residues at positions *a* and *d*. Each layer is formed by interactions among three side chains of *a-a'-a"* or *d-d'-d"*. The core interaction for the 2nd heptad is exemplified: Leu9-Leu9-Leu9 (dotted purple line) and Leu12-Leu12-Leu12 (dotted green line). **B.** Cross-sectional view of the 2nd heptad layer in the apo-GRW-L16CL30H 3SCC model. The model was generated from the crystal structure of PDB 7N2Y,[13] with residues in the heptad repeat mutated to match the sequence of GRW-L16CL30H.

Expected structural features of a putative antiparallel GRW-L16CL30H assembly

We have previously shown that heme binding to apo-GRW-L16CL30H is accompanied by a change in oligomerization state and topology (from a parallel 3SCC to an antiparallel (heme)$_2$4SCC assembly).[16] It was also capable of reversibly modulating cofactor coordination and spin state in response to changes in pH within this single minimal scaffold. The heme was shown to bind as a 5-coordinate imidazole complex between pH values 7-9 and then converted first to a high spin, 5-coordinate thiolate species until pH 10 at which time a low spin, 6-coordinate thiolate compound was generated.[15,16] These different coordination modes provide active-site mimics of peroxidases, chloroperoxidases, and cytochrome P450 monooxygenases. Therefore, having established the canonical structural features and interhelical interactions of the parallel apo-3SCC, we next considered how changes in oligomeric state and helix orientation would influence coiled coil packing.

For such a topology, AlphaFold 3 fails to generate a satisfactory structural model for either an antiparallel apo-GRW-L16CL30H 4SCC or the corresponding heme-bound (heme)$_2$4SCC. Nevertheless, by referencing previously reported canonical antiparallel 4SCC crystal structures,[17–19] the (heme)$_2$4SCC assembly is expected to form an ABAB arrangement with a hydrophobic core composed of two alternating sets of *a-d-a′-d′* and *d-a-d′-a′* interactions extending along the coiled coil (Fig. S14). These distinct packing arrangements place the potentially coordinating residues, Cys16 and His30, in different structural environments, generating mixed layers of the type Leu_2Cys_2 and Leu_2His_2. For GRW-L16CL30H, an antiparallel configuration would position Lys residues on adjacent *g-g′* faces and Glu residues on adjacent *e-e′* faces, creating an unfavorable electrostatic environment that would preclude charge-pair contacts and eliminate the additional interhelical stabilization typically observed in canonical coiled coils.[20] In such a context, the formation of the antiparallel assembly is likely driven by alternative stabilizing factors, most prominently heme binding at the interface of the dimers.

In some reported cases,[19] the flexibility of residues such as Lys allows the terminal ammonium groups to adopt conformations that avoid direct charge-charge repulsion and instead form salt bridges with alternative partners. Bridging water molecules between like-charged residues may further help alleviate electrostatic repulsion. Minor adjustments in helix register and side-chain conformation to accommodate both the hydrophobic core and the electrostatic patterning could similarly be envisioned for the (heme)$_2$4SCC assembly; however, in the absence of a crystal structure or a satisfactory prediction model, this hypothesis cannot be directly confirmed.

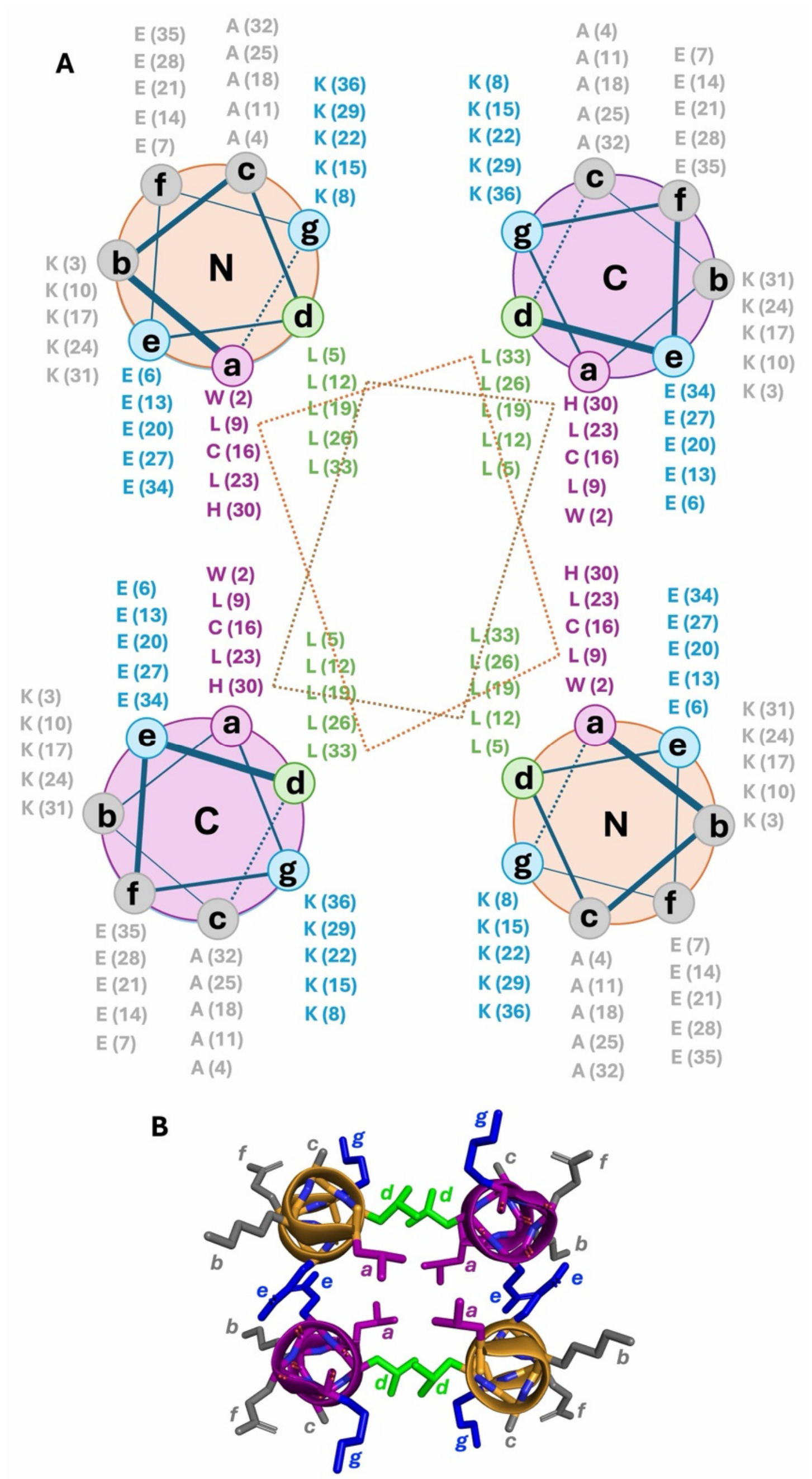

Figure S14. Helical wheel diagram for antiparallel 4SCC GRW-L16CL30H. A. Two peptide helices (light orange) extend from the N-terminus (emerging from the page) to the C-terminus (projecting into the page), while the other two (light purple) extend from the C-terminus (emerging from the page) to the N-terminus (projecting into the page) to form an ABAB arrangement. Each hydrophobic layer is formed by interactions among four hydrophobic side chains of *a*-*d*-*a'*-*d'* or *d*-*a*-*d'*-*a'*. The core interaction for the 2nd heptad (from the N-terminal) is exemplified: Leu9-Leu33-Leu9-Leu33 (dotted orange line) and Leu12-His30-Leu12-His30 (dotted brown line). Considering a canonical antiparallel 4SCC with an *a/d* hydrophobic core, no interhelical salt bridges are allowed to be formed due to charge repulsion between *e*-*e'* and g-*g'* positions. **B.** Cross-sectional view of the 2nd heptad layer in the GRW-L16CL30H 4SCC model. The model was generated from the crystal structure of PDB 8A3G,[18] with residues in the heptad repeat mutated to match the sequence of GRW-L16CL30H.

References

(1) Zastrow, M. L.; Pecoraro, V. L. Designing Functional Metalloproteins: From Structural to Catalytic Metal Sites. *Coord. Chem. Rev.* **2013**, *257* (17–18), 2565–2588. https://doi.org/10.1016/j.ccr.2013.02.007.

(2) Chakraborty, S.; Iranzo, O.; Zuiderweg, E. R. P.; Pecoraro, V. L. Experimental and Theoretical Evaluation of Multisite Cadmium(II) Exchange in Designed Three-Stranded Coiled-Coil Peptides. *J. Am. Chem. Soc.* **2012**, *134* (14), 6191–6203. https://doi.org/10.1021/ja210510g.

(3) Iranzo, O.; Cabello, C.; Pecoraro, V. L. Heterochromia in Designed Metallopeptides: Geometry-Selective Binding of Cd^{II} in a De Novo Peptide. *Angew. Chem. Int. Ed.* **2007**, *46* (35), 6688–6691. https://doi.org/10.1002/anie.200701729.

(4) Neupane, K. P.; Pecoraro, V. L. Probing a Homoleptic PbS_3 Coordination Environment in a Designed Peptide Using ^{207}Pb NMR Spectroscopy: Implications for Understanding the Molecular Basis of Lead Toxicity. *Angew. Chem. Int. Ed.* **2010**, *49* (44), 8177–8180. https://doi.org/10.1002/anie.201004429.

(5) Matzapetakis, M.; Farrer, B. T.; Weng, T.-C.; Hemmingsen, L.; Penner-Hahn, J. E.; Pecoraro, V. L. Comparison of the Binding of Cadmium(II), Mercury(II), and Arsenic(III) to the de Novo Designed Peptides TRI L12C and TRI L16C. *J. Am. Chem. Soc.* **2002**, *124* (27), 8042–8054. https://doi.org/10.1021/ja017520u.

(6) Matzapetakis, M.; Ghosh, D.; Weng, T.-C.; Penner-Hahn, J. E.; Pecoraro, V. L. Peptidic Models for the Binding of Pb(II), Bi(III) and Cd(II) to Mononuclear Thiolate Binding Sites. *JBIC Journal of Biological Inorganic Chemistry* **2006**, *11* (7), 876–890. https://doi.org/10.1007/s00775-006-0140-7.

(7) Borghesani, V.; Zastrow, M. L.; Tolbert, A. E.; Deb, A.; Penner-Hahn, J. E.; Pecoraro, V. L. Co(II) Substitution Enhances the Esterase Activity of a de Novo Designed Zn(II) Carbonic Anhydrase. *Chemistry – A European Journal* **2024**, *30* (24). https://doi.org/10.1002/chem.202304367.

(8) Pitts, W. C.; Deb, A.; Penner-Hahn, J. E.; Pecoraro, V. L. Revving up a Designed Copper Nitrite Reductase Using Noncoded Active Site Ligands. *ACS Catal.* **2024**, *14* (6), 4362–4368. https://doi.org/10.1021/acscatal.3c06159.

(9) Cangelosi, V. M.; Deb, A.; Penner-Hahn, J. E.; Pecoraro, V. L. A De Novo Designed Metalloenzyme for the Hydration of CO_2. *Angewandte Chemie* **2014**, *126* (30), 8034–8037. https://doi.org/10.1002/ange.201404925.

(10) La Gatta, S.; Firby, J. K.; Penner-Hahn, J. E.; Pecoraro, V. L. Probing Anion Recognition in a Cobalt(II) de Novo Designed Metalloprotein. *J. Inorg. Biochem.* **2026**, *279*, 113268. https://doi.org/10.1016/j.jinorgbio.2026.113268.

(11) Touw, D. S.; Nordman, C. E.; Stuckey, J. A.; Pecoraro, V. L. Identifying Important Structural Characteristics of Arsenic Resistance Proteins by Using Designed Three-Stranded Coiled Coils. *Proceedings of the National Academy of Sciences* **2007**, *104* (29), 11969–11974. https://doi.org/10.1073/pnas.0701979104.

(12) Zastrow, M. L.; Peacock, A. F. A.; Stuckey, J. A.; Pecoraro, V. L. Hydrolytic Catalysis and Structural Stabilization in a Designed Metalloprotein. *Nat. Chem.* **2012**, *4* (2), 118–123. https://doi.org/10.1038/nchem.1201.

(13) Pinter, T. B. J.; Ruckthong, L.; Stuckey, J. A.; Deb, A.; Penner-Hahn, J. E.; Pecoraro, V. L. Open Reading Frame 1 Protein of the Human Long Interspersed Nuclear Element 1 Retrotransposon Binds Multiple Equivalents of Lead. *J. Am. Chem. Soc.* **2021**, *143* (37), 15271–15278. https://doi.org/10.1021/jacs.1c06461.

(14) Lovejoy, B.; Choe, S.; Cascio, D.; McRorie, D. K.; DeGrado, W. F.; Eisenberg, D. Crystal Structure of a Synthetic Triple-Stranded α-Helical Bundle. *Science (1979).* **1993**, *259* (5099), 1288–1293. https://doi.org/10.1126/science.8446897.

(15) Modenez, I. A.; Ivancich, A.; Pecoraro, V. De Novo Design of α-Helical Heme Binding Proteins Capable of Versatile Cofactor Ligation; 2025; pp 115–139. https://doi.org/10.1016/bs.mie.2025.07.013.

(16) Koebke, K. J.; Kühl, T.; Lojou, E.; Demeler, B.; Schoepp-Cothenet, B.; Iranzo, O.; Pecoraro, V. L.; Ivancich, A. The PH-Induced Selectivity Between Cysteine or Histidine Coordinated Heme in an Artificial A-Helical Metalloprotein. *Angew. Chem. Int. Ed.* **2021**, *60* (8), 3974–3978. https://doi.org/10.1002/anie.202012673.

(17) Deng, Y.; Liu, J.; Zheng, Q.; Eliezer, D.; Kallenbach, N. R.; Lu, M. Antiparallel Four-Stranded Coiled Coil Specified by a 3-3-1 Hydrophobic Heptad Repeat. *Structure* **2006**, *14* (2), 247–255. https://doi.org/10.1016/j.str.2005.10.010.

(18) Naudin, E. A.; Albanese, K. I.; Smith, A. J.; Mylemans, B.; Baker, E. G.; Weiner, O. D.; Andrews, D. M.; Tigue, N.; Savery, N. J.; Woolfson, D. N. From Peptides to Proteins: Coiled-Coil Tetramers to Single-Chain 4-Helix Bundles. *Chem. Sci.* **2022**, *13* (38), 11330–11340. https://doi.org/10.1039/D2SC04479J.

(19) Yadav, M. K.; Leman, L. J.; Price, D. J.; Brooks, C. L.; Stout, C. D.; Ghadiri, M. R. Coiled Coils at the Edge of Configurational Heterogeneity. Structural Analyses of Parallel and Antiparallel Homotetrameric Coiled Coils Reveal Configurational Sensitivity to a Single Solvent-Exposed Amino Acid Substitution †. *Biochemistry* **2006**, *45* (14), 4463–4473. https://doi.org/10.1021/bi060092q.

(20) Woolfson, D. N. Understanding a Protein Fold: The Physics, Chemistry, and Biology of α-Helical Coiled Coils. *Journal of Biological Chemistry* **2023**, *299* (4), 104579. https://doi.org/10.1016/j.jbc.2023.104579.
